# Juvenile hormone regulation of Drosophila aging

**DOI:** 10.1186/1741-7007-11-85

**Published:** 2013-07-17

**Authors:** Rochele Yamamoto, Hua Bai, Adam G Dolezal, Gro Amdam, Marc Tatar

**Affiliations:** 1 Department of Ecology and Evolutionary Biology, Brown University, Providence, RI, USA; 2 School of Life Sciences, Arizona State University, Tempe, AZ, USA; 3 Current address: Department of Ecology, Evolution, and Organismal Biology, Iowa State University, Ames, IA, USA

**Keywords:** Juvenile hormone, Drosophila, Lifespan, Fecundity, Fat body, Gene expression

## Abstract

**Background:**

Juvenile hormone (JH) has been demonstrated to control adult lifespan in a number of non-model insects where surgical removal of the *corpora allata* eliminates the hormone’s source. In contrast, little is known about how juvenile hormone affects adult *Drosophila melanogaster*. Previous work suggests that insulin signaling may modulate Drosophila aging in part through its impact on juvenile hormone titer, but no data yet address whether reduction of juvenile hormone is sufficient to control Drosophila life span. Here we adapt a genetic approach to knock out the *corpora allata* in adult *Drosophila melanogaster* and characterize adult life history phenotypes produced by reduction of juvenile hormone. With this system we test potential explanations for how juvenile hormone modulates aging.

**Results:**

A tissue specific driver inducing an inhibitor of a protein phosphatase was used to ablate the *corpora allata* while permitting normal development of adult flies. *Corpora allata* knockout adults had greatly reduced fecundity, inhibited oogenesis, impaired adult fat body development and extended lifespan. Treating these adults with the juvenile hormone analog methoprene restored all traits toward wildtype. Knockout females remained relatively long-lived even when crossed into a genotype that blocked all egg production. Dietary restriction further extended the lifespan of knockout females. In an analysis of expression profiles of knockout females in fertile and sterile backgrounds, about 100 genes changed in response to loss of juvenile hormone independent of reproductive state.

**Conclusions:**

Reduced juvenile hormone alone is sufficient to extend the lifespan of *Drosophila melanogaster*. Reduced juvenile hormone limits reproduction by inhibiting the production of yolked eggs, and this may arise because juvenile hormone is required for the post-eclosion development of the vitellogenin-producing adult fat body. Our data do not support a mechanism for juvenile hormone control of longevity simply based on reducing the physiological costs of egg production. Nor does the longevity benefit appear to function through mechanisms by which dietary restriction extends longevity. We identify transcripts that change in response to juvenile hormone independent of reproductive state and suggest these represent somatically expressed genes that could modulate how juvenile hormone controls persistence and longevity.

## Background

Juvenile hormone (JH) plays a central role in insect development where it interacts with ecdysone to maintain larval *status quo* between molts [1]. In adults, JH takes on different functions. From work with a variety of insects, there is consensus that JH produced in adult *corpora allata* modulates ovarian maturation, in part through its regulation of yolk protein uptake while ecdysone derived from the follicle cells induces yolk protein synthesis in fat bodies [2-7]. JH in the adult also affects learning, migration, diapause and innate immunity [8-11]. In the monarch butterfly, some grasshoppers and the firebug *Pyrrhocoris apterus*, surgical allatectomy extends lifespan, suggesting that JH is a pro-aging hormone [12-14].

*Drosophila melanogaster* produces three forms of juvenile hormone: methyl farnesoate (MF), JHIII and bisepoxide JHIII (JHBIII) [15]. JHBIII is unique to dipterans and is the most abundant JH in flies. In other insects, exogenous JH or juvenile hormone analogs (JHA), such as methoprene, induce supernumerary juvenile or pupal stages and block metamorphosis, but Drosophila larvae treated with JH analogs still initiate metamorphosis [16,17], although high doses of JHA produce a mosaic adult where the abdomen is covered with pupal cuticle [18]. The high dosage required to induce this phenotype, and an artifact produced by a common genetic marker (*rosy* encoding xanthine dehydrogenase), have complicated our ability to study the roles of JH in Drosophila development [19,20]. Even less is known about the role of JH in the Drosophila adult, in part because treating wildtype adults with JH analogs produces few obvious phenotypes and because surgical allatectomy is not practical.

One potential role for JH in adult Drosophila became apparent in studies of fly insulin-like receptor mutants. Drosophila insulin-like receptor (*InR*) mutants have ovaries similar to those seen in wildtype flies during reproductive diapause [21]. Drosophila reproductive diapause is maintained in part by reduced JH [22,23], and insulin-like signaling mutants were found to be JH deficient [21]. *InR* mutants are long-lived, and JHA treatment restores lifespan toward that of wildtype flies. Together these observations motivated the question of our current study: Is JH deficiency sufficient to extend lifespan in Drosophila, and if so, by what mechanism?

JH production in Drosophila might be experimentally reduced in several ways. A classic genetic approach used the *apterous* mutant, which regulates development in wing, muscle and axons, and neuroendocrine control of juvenile hormone as well as other neuropeptides [24-28]. More recently, the P{Gal4} insertion (*Aug21*) was found to be expressed in larval *corpora allata*[29] and Riddiford *et al.*[30] used *Aug21* to drive UAS-*grim* to induce apoptosis in larval *corpora allata* (CA). Allatectomized larvae produced small pupae that died at head eversion and showed many photoreceptor development defects. Liu *et al.*[31] used this genotype to reveal how the JH-interacting proteins MET (methoprene-tolerant) and GCE (Germ-cell expressed bHLH-PAS) regulate larval fat body programmed cell death. To study adult traits, Gruntenko *et al.*[32] drove CA apoptosis with a combination of *UAS-reaper* and *UAS-hid*. Unlike *Aug21* > *UAS-grim*, this genotype produced viable adults and showed impaired JH metabolism, decreased resistance to heat stress, and reduced female fecundity. This genetically mediated CA-ablation also increased the level of dopamine in young females, accompanied by decreases in the activity of enzymes responsible for both the synthesis and degradation of this amine [33].

Here, we modified these approaches to study how aging is affected by allatectomy that reduces endogenous JH in adult Drosophila. We used *Aug21* to over-express *Nuclear Inhibitor of Protein Phosphatase type 1* (*NIPP1*) [34] and thus eliminate the CA of newly eclosed adults. From these adults and their coisogenic controls, we measured lifespan and tested potential mechanisms by which reduced JH could contribute to longevity assurance. Allatectomy reduced egg production as expected, and fecundity was partially restored by treating adults with exogenous JHA. Reduced JH extended lifespan in fertile flies and, importantly, it also did so in adults made sterile by the dominant *Ovo*^*D1*^ mutation, which inhibits egg maturation [35]. These data suggest that reduced JH extends lifespan by mechanisms independent of direct physiological trade-offs with egg production. Allatectomy extended lifespan robustly in flies fed different concentrations of yeast, eliminating a potential explanation based on mechanisms of dietary restriction. Finally, we analyzed gene expression profiles from allatectomized adults when fertile and when *Ovo*^*D1*^ sterile, and from the intersection of these data identified soma-related genes through which JH may potentially modulate longevity.

## Results and discussion

### *Corpora allata* knockout (CAKO)

**Figure 1:**
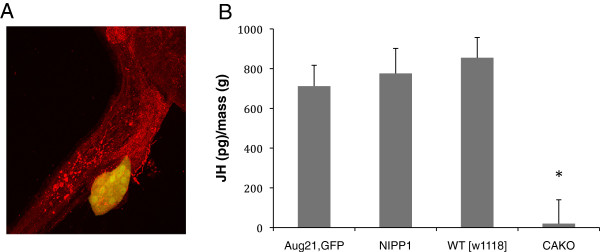
***Corpora allata *knockout of adult Drosophila reduces juvenile hormone. A)** Adult *corpora allata* (yellow cells) marked by *Aug21-Gal4* driving *UAS-GFP* (*Aug21, GFP*). **B)** Total JH measured from adults by GC-MS per wet mass (total body). Genotypes with intact *corpora allata*: *Aug21, GFP*; *UAS-NIPP1*, wildtype (WT, [*w*^*1118*^]). *Corpora allata* knockout (CAKO): *Aug21,GFP*; *UAS-NIPP1*. Error bars are standard deviation; significant difference indicated by * (ANOVA, *P* < 0.000,1).

JH is normally abundant in females, at least up to age 10 days [36]. Here, the genotype *Aug21-Gal4, UAS-GFP* >*UAS-NIPP1* (CAKO: *Corpora Allata* Knock Out) produced adults within 12 to 24 hours post-eclosion with small to non-existent *corpora allata* and limited levels of JH as measured by gas chromatography–mass spectrometry. Females of wildtype and parental genotypes contained between 700 and 850 pg JH per gram body mass while CAKO females had less than 50 pg/g (Figure 1).

### CAKO reduces fecundity and impedes adult fat body development

**Figure 2:**
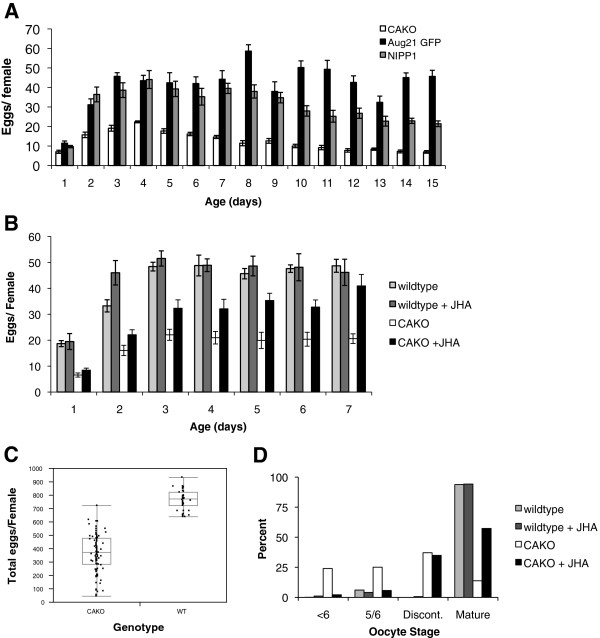
**Egg production in response to *corpora allata *knockout and rescue by JHA. A)** Daily per capita fecundity of mated females in cages. Control genotypes with intact *corpora allata*: *Aug21, GFP* and *UAS-NIPP1*. *Corpora allata* knockout (CAKO): *Aug21,GFP*; *UAS-NIPP1*. **B)** Daily per capita fecundity of mated females in cages: wildtype (*w*^*1118*^) and CAKO mated females exposed to vehicle control (EtOH) or to methoprene (JHA). In **(A)** and **(B)** error bars are standard deviation. **C)** Total eggs per mated, individually held females (age 2 to 10 days). Whisker plots show individual values, group means (wildtype, 764 eggs; CAKO, 365 eggs), one standard deviation (wildtype, 74 eggs; CAKO, 140.6 eggs) and range. **D)** Distribution of ovariole maturation scored by oocyte stage from mated females aged 10 days; wildtype and CAKO females, exposed to vehicle or JHA. The percentage is among ovarioles of one ovary, averaged among 842 (wildtype no JHA), 71 (wildtype with JHA), 849 (CAKO no JHA) and 780 (CAKO with JHA) females per group. Oocyte stage categories: <6, the most mature egg chamber in ovariole has no yolk; 5/6, the most mature egg chamber in ovariole has little yolk; Discont. (discontinuous), ovariole contains one mature (or hypertrophied mature) egg chamber but next most mature egg chamber contains no yolk (stage <6); Matureovariole has a continuous sequence of mature and maturing egg chambers with at least one chamber between stage 7 and 12.

CAKO strongly reduced egg production. When measured from caged cohorts, average daily fecundity of control genotypes peaked at 50 to 60 eggs per female, while CAKO females laid less than 20 eggs per day (Figure 2A). Fecundity was also measured from individually maintained females (Figure 2C). From eclosion to age 10 days, wildtype females produced two-fold more eggs than CAKO. Notably, the variance in total egg production was two-fold greater in CAKO, suggesting there were underlying differences in the efficiency of *corpora allata* knockout. Thus, when measured from a collection of caged adults, the net sample of CAKO females may yield some eggs because a few females retain sufficient levels of JH. Individually held CAKO females show that some individuals indeed produce nearly wildtype quantities of eggs while some produce very few eggs. In the cases where eggs are produced, these females may have initiated these eggs before the CA-ablation was complete, retained enough residual JH after the ablation-period to mature some eggs, or have the capacity to produce eggs in the absence of JH. We cannot distinguish between these alternatives because measuring JH levels requires destructive samples comprised of many individuals. To test if reduced fecundity was caused specifically by the loss of JH in allatectomized females we measured daily fecundity in adults exposed to methoprene (JHA). As previously reported [32], JHA did not affect egg production in wildtype females but JHA increased egg production in allatectomized females (Figure 2B). Notably, complete rescue of daily fecundity was statistically achieved only after seven days of JHA treatment, suggesting that the loss of JH in CAKO impairs some aspect of post-eclosion ontogeny that takes some time to restore.

**Figure 3:**
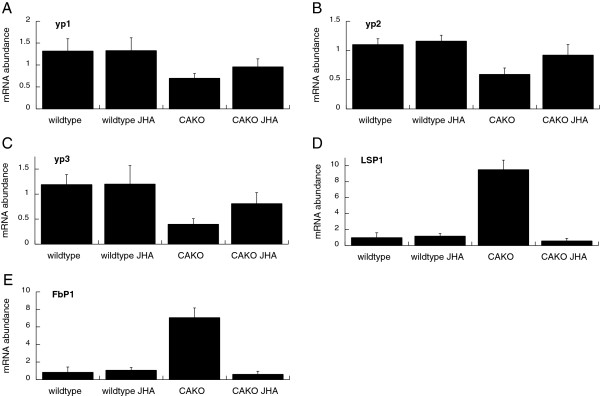
**mRNA abundance for fat body related genes.** All plots: wildtype (*w*^*DAH*^/*w*^*1118*^) relative to CAKO, and both genotypes exposed to vehicle (genotype name alone in label) or JHA; **A-C)** Yolk proteins (*yp1*, *yp2*, *yp3*), **D)** larval serum protein (*LSP1*), **E)** fat body protein 1 (*FbP1*). Error bars show 95% confidence intervals among three biological replicates.

In many insects, including Drosophila, JH modulates fecundity at least in part because the hormone is required to induce yolk proteins uptake into oocytes [37,38], while ecdysone produced from egg follicles induces yolk protein mRNA expressed in the fat body [2,3,7]. We found yolk protein mRNA (*yp1*, *yp2*, *yp3*) was reduced in CAKO females while treatment of these flies with JHA partially restored their yolk protein expression (Figure 3A-C). Likewise, wildtype females had mature ovarioles that contained previtellogenic egg chambers at stages 1 to 6, one or two yolk-containing egg chambers (stages 7 to 14) and a mature egg (Figure 2D). In contrast, ovarioles from CAKO females contained only previtellogenic egg chambers, or they were ‘discontinuous’ because they contained previtellogenic chambers with one mature egg. JHA treatment increased the proportion of mature ovarioles in CAKO females but did not affect the distribution of ovariole types in wildtype females. Together these data suggest that CAKO females have reduced fecundity because they have limited yolk protein. The discontinuous quality of CAKO ovarioles may arise because newly eclosed females possess enough JH at eclosion to initiate one mature egg but with allatectomy they cannot sustain vitellogenesis.

Adult fat body is a major site of yolk protein synthesis. At emergence, Drosophila adults contain larval fat body [39]. This larval fat body undergoes histolysis in 24 to 48 hours in wildtype adults, while adult fat body develops from progenitor cells. Postlethwait and Jones [40] recognized that JH plays a role in larval fat histolysis. Here, we evaluated fat body status in 15-day-old females. Among 40 wildtype females all but one exclusively contained adult fat body. Among 91 CAKO females, 51 retained some larval fat body while 40 contained only adult fat body. On the other hand, 28 of 30 CAKO females treated with JHA contained only adult fat body. CAKO adults also expressed mRNA from genes that are characteristic of larval fat body, including larval serum protein 1 (*LSP1α*) and fat body protein 1 (*FbP1*) (Figure 3D,E). These larval-associated mRNA were repressed when CAKO females were treated with JHA soon after eclosion. The impaired fecundity and low expression of yolk protein mRNA of CAKO adults may arise in part because they delay full development of adult fat bodies. Treating CAKO adults with JHA induces the transition from larval to adult fat body and may thus facilitate yolk protein production and fecundity.

### CAKO extends lifespan independent of egg production

Surgical allatectomy extends lifespan in desert grasshoppers, monarch butterflies and the firebug *Pyrrhocoris apterus*[12-14]. In each case, life expectancy of adults treated with JH or JHA is reduced to that of sham-operated adults. While these results are robust, little is known about the mechanisms by which JH affects lifespan. Drosophila provides a tractable system to study potential mechanism, and CAKO offers a useful platform to describe how loss of JH modulates adult Drosophila lifespan.

**Figure 4:**
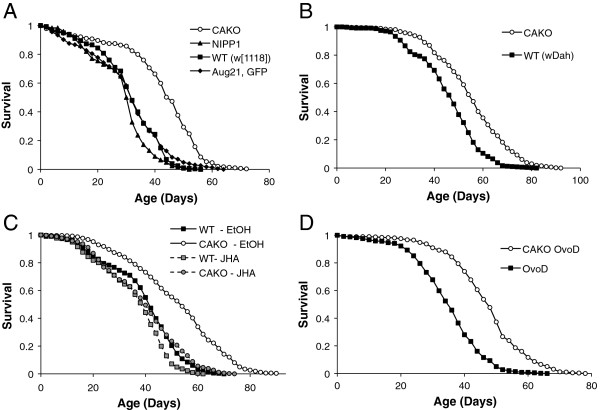
**Adult survival of control and CAKO genotypes, and response to JHA. A)** CAKO (in *w*^*1118*^ background) relative to three control genotypes with intact *corpora allata* (*Aug21, GFP*; *UAS-NIPP1*, wildtype (WT(*w*^*1118*^)). **B)** CAKO (in *w*^*DAH*^ background) relative to wildtype (*w*^*DAH*^). **C)** CAKO (in *w*^*DAH*^*/w*^*1118*^ background) exposed to vehicle (EtOH) relative to CAKO treated with JHA, and compared to wildtype (*w*^*DAH*^*/w*^*1118*^) with EtOH or JHA. **D)** CAKO (in *w*^*DAH*^/*w*^*1118*^ background) with *Ovo*^*D1*^ mutation relative to control *w*^*DAH*^/*w*^*1118*^ with *Ovo*^*D1*^ mutation; females of both cohorts produce no eggs. In each panel, CAKO overall mortality is significantly less than each control (log rank test, *P* < 0.0001) while there are no significant differences among controls within a trial.

The *Aug21,GFP* and *NIPP1* stocks were introgressed into two wildtype backgrounds. Survival of female offspring with the *Aug21,GFP; NIPP1* (CAKO) genotype were then compared to otherwise coisogenic parental genotypes and wildtype stocks (life table statistics in Additional file 1: Table S1). In the *w*^*1118*^ background, CAKO increased adult survival relative to all genetic controls (Figure 4A) by consistently reducing age-specific adult mortality (Additional file 2: Figure S1). Female longevity was likewise extended when CAKO was introgressed into the *white*-Dahomey (w^DAH^) wildtype background (Figure 4B, Additional file 2: Figure S1). Although the role of JH in males is generally enigmatic, CAKO was also sufficient to extend male lifespan (Additional file 3: Figure S2).

To establish whether loss of JH upon allatectomy is sufficient to extend lifespan we exposed control and CAKO adult females to the JHA methoprene (Figure 4C). Longevity was extended in CAKO adults exposed to vehicle relative to similarly treated wildtype controls. Survival of CAKO flies exposed to JHA was reduced to that of wildtype flies with or without JHA. As seen with surgically allatectomized insects, the longevity benefit from genetically ablated CA in Drosophila is reversed by JHA. The observed gain in lifespan appears to be specifically caused by the loss of JH in CAKO adults.

Longevity is extended in many animals when fecundity is experimentally repressed, presumably by preventing physiological trade-offs between survival and egg production [41-43]. Reduced egg production is also a feature of insect reproductive diapause, a state induced in part by reduced JH synthesis [11,22,44]. Reproductive diapause slows aging in *Drosophila melanogaster* as it does in the endemic *D. triauraria* (Japan) and *D. littoralis* (Finland) [23,45,46]. Eliminating reproductive trade-offs may account for the increased longevity of CAKO. To test this hypothesis we backcrossed *Ovo*^*D1*^ into *w*^*1118*^ and generated *Ovo*^*D1*^; *Aug21, GFP*. *Ovo*^*D1*^ dominantly represses egg maturation by autonomously blocking oocyte progression at stages 2 and 3 [35]. This mutation has been used previously to demonstrate that Drosophila longevity assurance produced by dietary restriction is not caused by reduction in egg maturation [47]. Importantly, our data show that CAKO arrests oogenesis at the pre-vitellogenic check-point of stages 6 to 7 [37,38]. Both *Ovo*^*D1*^ and CAKO; *Ovo*^*D1*^ females are sterile because they arrest oocytes at stages 2 to 3; the oocyte arrest of *Ovo*^*D1*^ supersedes the later stage arrest of CAKO. In this way, both genotypes similarly avoid survival costs of reproduction associated specifically with egg production. All the same, sterile CAKO; *Ovo*^*D1*^ females remained long-lived relative to sterile *Ovo*^*D1*^ controls (Figure 4D). Avoiding costs of egg production does not fully account for the mechanism by which CAKO extends lifespan.

### CAKO independent of reproduction modulates somatically related genes

**Figure 5:**
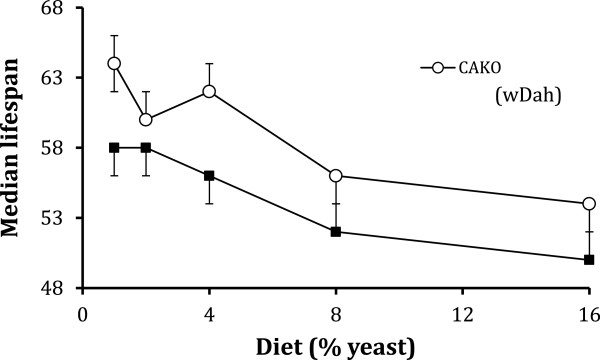
**Adult survival in response to dietary restriction.** CAKO and wildtype in *w*^*1118*^/*w*^*DAH*^ background. Median lifespan of female cohorts (upper and lower 95% confidence interval) maintained on sugar-cornmeal diets with yeast at 1%, 2%, 4%, 8% and 16%. Relative risk ratios (Cox proportional hazard mortality) do not statistically differ among flies at 2%, 8% and 16%, or among those at 1% and 4% (Additional file 1).

These results suggest that JH controls aging to some extent because it directly affects mechanisms of somatic survival. Dietary restriction is one process that may somatically control Drosophila longevity independent of egg production [47]. To test if reduced JH and dietary restriction mediate lifespan through common mechanisms, we measured lifespan of adults maintained on five diets ranging from 1.0% to 16% yeast. Median lifespan was increased in both wildtype and CAKO adults when the quantity of dietary yeast was reduced. The lifespan of CAKO was greater than that of wildtype at every diet level (Figure 5), and the magnitude of mortality differences between these genotypes was similar across all diets (Additional file 1: Table S1). Dietary restriction and CAKO thus appear to affect longevity through independent mechanisms.

**Figure 6:**
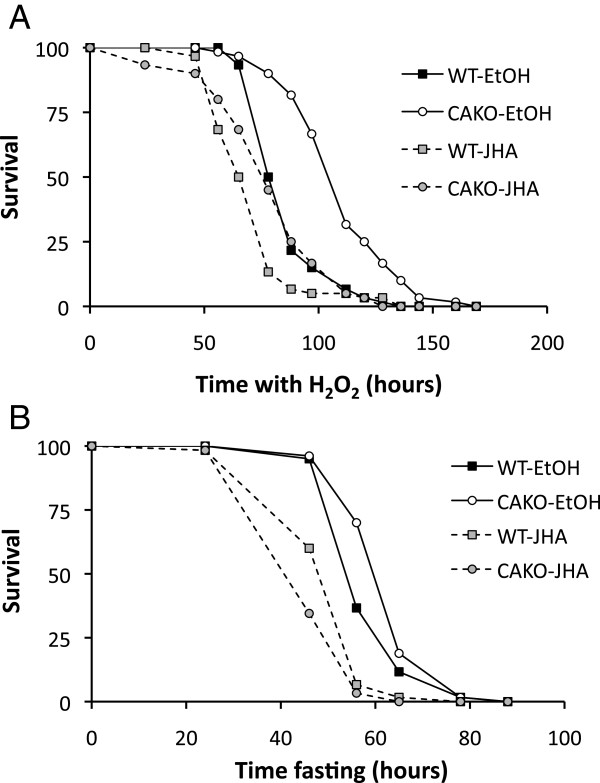
**Adult female stress survival.** CAKO and *w*^*1118*^/*w*^*DAH*^wildtypecontrol, treated with vehicle (EtOH) or JHA while **A)** exposed to hydrogen peroxide, or **B)** fasted.

Extended longevity in Drosophila is often associated with resistance to exogenous stress [48]. Resistance to heat and oxidative stress is also a feature of Drosophila reproductive diapause, and this trait is blunted when adults are treated with JHA [23]. Gruntenko *et al*. [32] found allatectomized females, but not males, were resistant to heat stress. Here we see that CAKO extended survival in females exposed to H_2_O_2_ and this capacity was reduced when adults were simultaneously treated with JHA (Figure 6A). CAKO, however, did not increase survival when flies were starved, although starvation survival was reduced when CAKO and wildtype females were treated with JHA (Figure 6B).

**Figure 7:**
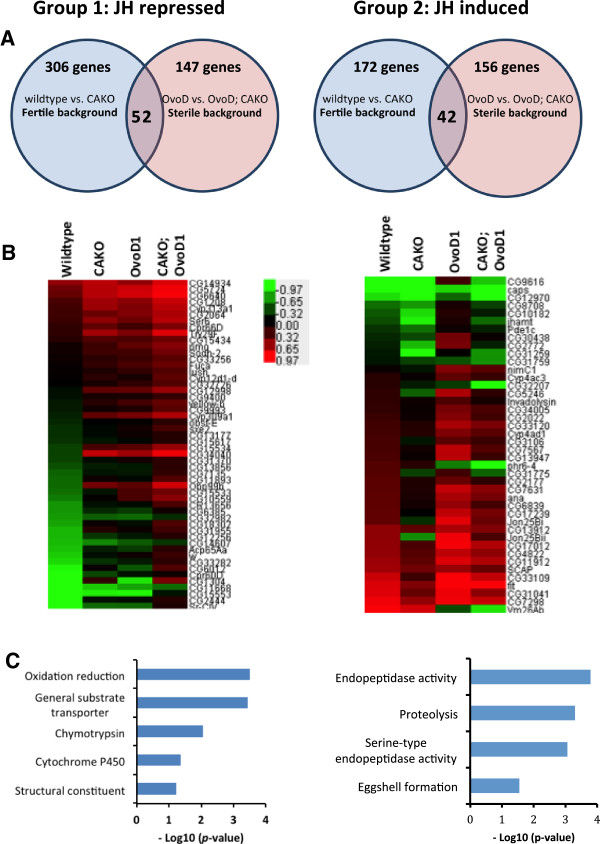
**mRNA abundance from females with and without *corpora allata*, and interactions with dominant sterile*Ovo***^***D1***^**.** CAKO and wildtype are in *w*^*DAH*^ background. *Ovo*^*D1*^ and CAKO; *Ovo*^*D1*^ are in *w*^*1118*^/*w*^*DAH*^ background. **A)** Group 1; genes repressed by JH: mRNA of 52 genes induced in females without *corpora allata* (CAKO) in both the fertile and sterile (*Ovo*^*D1*^) genotypes. Group 2; genes induced by JH: mRNA of 42 genes decreased in females without *corpora allata* (CAKO) in both the fertile and sterile (*Ovo*^*D1*^) genotypes. **B)** Heat map of overlap genes within each group. **C)** Gene Ontology for most enriched categories within each group.

CAKO thus appears to extend lifespan directly through effects upon somatic physiology, stress resistance or homeostasis rather than indirectly by avoiding costs of egg production. To identify somatic processes affected by JH independent of egg production, we compared gene expression profiles of CAKO females to expression profiles of wildtype controls, and we compared CAKO; *Ovo*^*D1*^ females to sterile *Ovo*^*D1*^ (intact CA) controls. These overlapping sets revealed 94 genes with changes correlated to longevity assurance but independent of reproductive state (Figure 7A,B). The genes are categorized into two groups. Group 1 includes transcripts that increase in CAKO and reflect genes that are normally repressed by JH in wildtype flies (Figure 7: JH repressed) (Additional file 4: Table S2). The induction of these genes is positively correlated with longevity extension; these genes thus contain candidates that confer longevity assurance. Group 2 includes transcripts that decrease in CAKO and thus reflect genes that are otherwise induced in animals with intact *corpora allata* (Figure 7: JH induced) (Additional file 4: Table S2). Group 2 genes reflect genes that are associated with pro-aging processes. Besides representing different individual genes, these groups also differ in their biological functions represented through Gene Ontology (Figure 7C) where genes induced by JH involve processes of protein digestion or catabolism while those repressed by JH are statistically associated with oxidative reduction and substrate transport.

**Figure 8:**
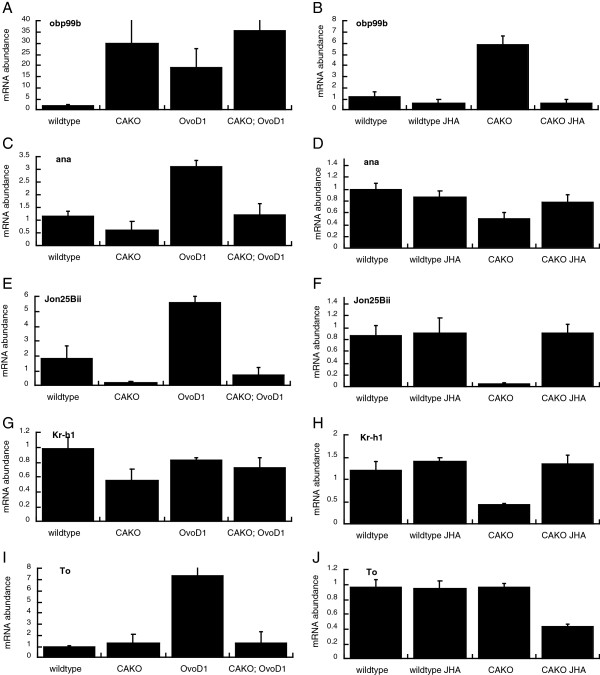
**mRNA abundance via qPCR for genes responsive to CAKO and JHA.** Left column: CAKO and wildtype in *w*^*1118*^/*w*^*DAH*^ background, *Ovo*^*D1*^ and CAKO; *Ovo*^*D1*^ in *w*^*1118*^/*w*^*DAH*^ background. Right column:wildtype (*w*^*1118*^/*w*^*DAH*^) relative to CAKO; exposed to vehicle (genotype name alone in label) or JHA. **A**, **B) ***Odorant binding protein 99b* (*Obp99b*). **C**, **D) ***Anachronism* (*ana*). **E**, **F) ***Johan 25Bii* (*Jon25Bii*). **G**, **H) ***Krupple homolog 1* (*Kr-h1*). **I**, **J) ***Takeout* (*to*). Error bars show 95% confidence intervals.

To validate this analysis for a sample of genes, we conducted real time quantitative PCR from CAKO and wildtype females in both sterile (*Ovo*^*D1*^) and fertile genetic backgrounds, and when fertile flies were treated with JHA (Figure 8). From Group 1, we tested *Obp99b*. mRNA of *Obp99b* was increased by allatectomy in both reproductive states (Figure 8A), and this expression was repressed when females were treated with JHA (Figure 8B). *Obp99b* is an odorant binding protein expressed in antennae and in fat body, and its expression was previously identified as JH-responsive in allatectomized larvae [31]. Some odorant receptor proteins can modulate Drosophila aging; mutants of *Orb83b* extend lifespan and enhance stress resistance [49]. *Obp99b* was also independently identified as a candidate gene to affect longevity in a mapping analysis of wildtype Drosophila [50].

Among genes induced by JH and associated with a pro-aging state (Group 2), we confirmed the expected mRNA responses for *Jon25Bii* and *ana* (Figure 8C-F). The gene *anachronism* (*ana*) encodes a secreted glycoprotein that participates in neuroblast development, neuromuscular function and wing development [51]. *Jon25Bii* is a predicted endopeptidase expressed abundantly in the midgut and is associated with Drosophila immune and xenobiotic responses [52,53]. *Jon25Bii* and *ana* have been identified as factors expressed in a fly model of mitochondrial disease [54]; they may contribute to aging by facilitating mitochondria functions that reduce survival [55].

Besides factors in these overlap groups, several genes of special interest changed in response to CAKO but only in one reproductive condition (Additional file 5: Table S3). *Kruppel homolog 1* (*Kr-h1*) is a JH-regulated zinc-finger transcription factor where it functions in processes such as metamorphosis, neurogenesis and foraging behavior [56-59]. In our arrays and confirmatory qPCR, mRNA for *Kr-h1* decreased in CAKO females of the fertile background exclusively, and this response was reversed by JHA treatment (Figure 8G,H). The gene *takeout* is a small peptide with sequence similarity to *Manduca sexta* juvenile hormone binding protein [60,61], although functional studies in Drosophila have yet to confirm JH binding for the Takeout protein. Bauer *et al*. [62] report that *takeout* is up-regulated in experimental conditions that extend Drosophila lifespan, and transgenic over-expression of *takeout* is sufficient to extend lifespan. The longevity benefit of *takeout* overexpression is repressed when flies are treated with JHA (methoprene), and JHA increases *takeout* mRNA in wildtype flies [60]. In our analysis, however, (Figure 8I,J), mRNA of *takeout* does not differ among wildtype and CAKO, but it is strongly elevated in *Ovo*^*D1*^, and this difference is eliminated in allatectomized *Ovo*^*D1*^ females. We find no effect of JHA treatment upon *takeout* mRNA abundance in either wildtype or CAKO genotypes. The regulation of *takeout* by JH appears to be complex and must depend on uncontrolled factors that differ among these studies.

**Figure 9:**
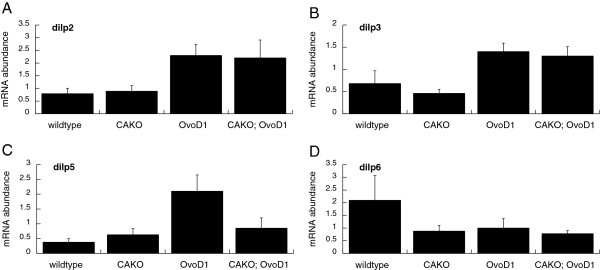
**mRNA abundance of Drosophila insulin-like peptides (*dilp*) in response to CAKO while interacting with*Ovo***^***D1***^**.** CAKO and wildtype are in *w*^*1118*^/*w*^*DAH*^ background. *Ovo*^*D1*^ and CAKO; *Ovo*^*D1*^ are in a *w*^*1118*^/*w*^*DAH*^ background. **A) ***dilp2*. **B) ***dilp3*. **C) ***dilp5*. **D) ***dilp6*. Error bars show 95% confidence intervals.

Ubiquitously expressed mutations of the insulin/IGF signaling system in Drosophila extend lifespan and reduce the production of JH from adult *corpora allata*[21,36]. On the other hand, an inverse interaction between JH and insulin signaling was seen in *Tribolium castaneum* where reduced JH promoted expression of insulin-like peptides [59]. Here we see that eliminating adult JH in Drosophila does not affect abundance of *dilp2* or *dilp3* mRNA (Figure 9A,B). Abundance of *dilp5* mRNA decreases with CAKO in the sterile *Ovo*^*D1*^ background, but not in fertile flies (Figure 9C). In contrast, *dilp6* mRNA declines strongly in CAKO adults in the fertile background, while *dilp6* mRNA is already low in all sterile *Ovo*^*D1*^ females. *dilp6* is predominantly expressed in adult fat body unlike neuron-expressed *dilp2*, *dilp3* and *dilp5*[63]. Overall, these data suggest that the impact of JH on aging is distal to longevity-regulatory *dilp2,* while JH contributes to the expression of *dilp6*, perhaps through its control of adult fat body.

## Conclusions

Juvenile hormone is an integrator of insect development, physiology and life history. In adult insects JH modulates alternative states of active reproduction versus dispersal, reproductive diapause and torpor [8]. JH supports the reproductive state in part by inducing fatty acid oxidation [64], and yolk protein uptake [4,5,7]. In contrast, JH is reduced during adult reproductive diapause or torpor. Such adults can be migratory [63], express lipogenic enzymes, accumulate lipids and triglycerides [64,65], resist exogenous stress, and live a relatively long time [12,46,66]. Many features of these syndromes appear in Drosophila, where adults in reproductive diapause have reduced titers of JH and increased triglycerides, glycogen, stress resistance and lifespan [22,23,67-70]. Our current work establishes that reduced JH is sufficient to extend Drosophila lifespan. Allatectomy increases Drosophila lifespan and this survival benefit is reduced by treatment with JHA. Although allatectomy also reduces fecundity in a JHA sensitive manner, the longevity of allatectomized adults is still expressed in otherwise sterile females. At a physiological level, JH appears to independently modulate egg production and survival. Under some ecological conditions a trade-off may occur between these fitness traits, and in this way selection would favor juvenile hormone-mediated pleiotropy between reproduction and aging. Hodkova [14] makes a similar argument for *Pyrrhocoris apterus* where surgical allatectomy extends lifespan in both fertile and surgically sterilized females*.* Our microarray analysis demonstrates there are at least some direct somatic physiological targets of JH that may be independent of its effects upon reproduction. This gene set provides a platform for hypotheses on how JH may mechanistically control longevity assurance.

## Methods

### Drosophila strains and culture

*UAS*-*NIPP1* (LA346) was provided by L. Alphey (Oxford University, Oxford, UK). The wildtype stock *w*^DAH^ was provided by the laboratory of L. Partridge (University College London, UK). *Aug21-Gal4* (*w*[*]; P{*w*[+mW.hs] = GawB}*Aug21/CyO*), *w*^1118^, *Ovo*^*D1*^ (*Ovo*^*D1*^*v*^*24*^/C(1)DX, *y*^*1*^*w*^*1*^*f*^*1*^) and *UAS-GFP* were obtained from Bloomington Stock Center, Indiana University, Bloomington, IN, USA. All UAS- and Gal4- lines in this study were backcrossed to *w*^DAH^ and *w*^1118^ backgrounds for six generations. The *Aug21-Gal4* and *UAS-GFP* were recombined to form the stock *Aug21,GFP.*

Flies were maintained at 25°C, 40% relative humidity and 12 h light:12 h dark. Stocks were reared at 25°C in standard lab food media (cornmeal (5.2%), sugar (11.0%), autolyzed yeast (2.5%; SAF brand, Lesaffre Yeast Corp., Milwaukee, WI, USA.), agar (0.79%) (w/v in 100 mL water) with 0.2% Tegosep (methyl4-hydroxybenzoate, Sigma, St Louis, MO, USA). Media for adults used this standard diet except with yeast concentrations as indicated at 1%, 2%, 4%, 8% and 16% w/v.

### *Corpora allata* ablation and juvenile hormone measure

*Corpora allata* (CA) were ablated by crossing female *UAS*-*NIPP1* to males of *Aug-21, GFP*. Flies were reared at 29°C until eclosion at which time they were maintained at 29°C as adults for two days (except as noted in the validation studies) and then transferred to 25°C for phenotype studies; these adults are designated as *Corpora Allata* Knock Out (CAKO).

To validate the efficiency of the tissue knockout we dissected control (15 of each sex) and CAKO (50 of each sex) adults, (both genotypes maintained at 29°C), at 24 hours post-eclosion to visualize the GFP signal at the *corpora allata*. Wildtype adults showed strong GFP florescence in large, full cells (as in Figure 1A) while all CAKO males and females either showed no (71 cases) or very faint (29 cases) florescence. To confirm how this reduction in *corpora allata* tissue affected its hormone secretion, we directly measured total juvenile hormone titers from whole adult females after 12 h at 29°C. A total of 25 replicates of 75 females for each genotype was collected at 12 h post eclosion and weighed (wet mass). Samples were added to 1.5 ml 90% HPLC grade methanol and frozen at −80°C until analysis. JH was then hexane extracted, column purified and converted to a d_3_-methoxyhydrin derivative. Total JH was then measured with a GC-MS coupled to mass selective detector/detection software (MSD/MDS) at Arizona State University [65,67]. Assays were conducted on an HP 6890 Series GC (Hewlett Packard, Palo Alto, CA, USA) equipped with a 30 m _ 0.25 mm CarbowaxEcono-Cap GC column (Alltech, Fresno, CA, USA) coupled to an HP 5973N inert mass selective detector/detection software (MSD/DS). This detection method provides high resolution between small quantities of the hormone. After purification and derivatization, JH form was identified by running analysis in SCAN mode and monitoring for known signatures of JHI (m/z 90 and 239), JHII (m/z 90 and 225), and JHIII (m/z 76 and 225) [66]. Subsequent samples were analyzed using the MSD/DS running in SIM mode, with monitoring of m/z 76 and 225 to insure specificity to JHIII. Total abundance was calculated against a standard curve for JHIII [65].

### Juvenile hormone analog treatment

Adult flies were exposed to the juvenile hormone analog methoprene (JHA) or EtOH vehicle control beginning at 12 h post eclosion and throughout the duration of each experiment, with fresh solutions provided every two days. Methoprene (Sigma: PESTANAL, racemic mixture) was diluted to 0.02 μg in 1 μl ethanol (100%). A total of 10 μl of solution was applied upon a cotton bud suspended in the experimental fly bottle or sealed demography cage.

### Lifespan

Newly eclosed adults were collected, transferred to bottles with fresh medium and kept at 29°C until 48 hours old. At 48 hours adults were sorted under light CO_2_ and transferred to demography cages maintained at 25°C at an initial density of 125 adults (single sex) per cage. Demography cages were 1L food service containers with a screen lid, aperture to remove flies and a passage-tube to a removable, standard food vial. Unless noted, food vials of demography cages contained standard sugar-cornmeal-2.5% yeast diet. Three to five replicate cages were initiated for every genotype or treatment cohort. Cages were maintained at 25°C with 40% relative humidity and 12 h light:12 h dark. Food was changed every two days, at which time dead flies were removed from each cage and counted.

Life tables were constructed by the extinct cohort method, combining data from all cages in a genotype or treatment group. Survival effects were analyzed within each experimental period using the log-rank test. Mortality rate (*μ*_x_) was calculated as ln(−ln(1-*q*_*x*_)) where *q*_*x*_ is age-specific mortality. Mortality rate was plotted for each cohort to confirm that survival differences reflect effects upon demographic aging as reflected by consistent differences in the progressive age-dependent risk of death.

### Stress assays

Five-day-old females were used to assess starvation and oxidative stress resistance. To assess starvation resistance, flies were transferred into glass vials containing 0.8% agar (with or without 0.1 μg methoprene/vial). Dead flies were counted twice a day. To assess oxidative stress resistance, flies previously maintained on regular media were transferred to glass vials containing 0.8% agar containing 5% sucrose and 5% H_2_O_2_ (with or without 0.1 μg methoprene/vial). Dead flies were counted twice a day. In both assays, about 80 females were distributed among eight test vials per treatment. Survival was analyzed by the log-rank test.

### Fecundity and ovariole staging

Fecundity of grouped females was measured across five replicate demography cages (20 females/cage) with 5 cm (dia) agar-plates attached to the terminal end of the food tubes. Females were housed with 20 wild-type *w*^*1118*^ males. Agar juice plates were supplemented with yeast paste and changed twice a day at which time eggs were counted. Dead females were removed and counted daily. Fecundity was calculated daily as total eggs/number living females, averaged across cages. Individual females were placed in vials with two *w*^*1118*^ males containing green-colored fly food and yeast paste. Each day, adult flies were changed to new vials and eggs were counted.

Ovariole stages were measured from females at 10 days old. Ovaries were dissected and fixed with 4% paraformaldehyde. Fixed ovarioles were separated with a stainless steel needle, and egg chamber stages were scored by the criteria of King [61].

### Microarray processing and analyses

Abundance of mRNA on a genomic scale was measured upon Affymetrix Drosophila 2.0 arrays. Adults from each genotype were aged to 14 days upon standard media in demography cages (125 females per cage, four cages per genotype). For each genotype, total RNA was isolated from 25 whole females with Trizol (Invitrogen, Life Technologies, Inc., Carlsbad, CA, USA.) and purified upon RNeasy columns (QIAGEN Inc., Valencia, CA, USA.). Using the Affymetrix One Cycle DNA system, 5 ug total RNA was converted to cDNA. Double stranded cDNA was amplified overnight at 37°C with the Affymetric IVT labeling protocol to produce biotin labeled cRNA. A total of 1.5 ug of spin column cleaned cRNA was fragmented by metal inducing hydrolysis, and 1.0 ug of this product was hybridized to arrays overnight at 45°C, 60 rpm. Hybridization, washing and staining were completed on an Affymetrix 450 Fluidics Station, and scanned on an Affymetrix 3000 G7. Three independent biological replicates were generated for each genotype.

Quantile normalized data were processed with Gene Chip Robust Multi-array Averaging (GC-RMA) [64] to generate log2 scaled expression values. Probesets with mean expression in the first quartile within a genotype were excluded. Two sided t-tests were used to assess differential expression with individual *P* < 0.05 and fold change at least 1.5. Probsets were collapsed to genes after differential expression statistics selection. All expression data from these arrays are publically available at NCBI GEO (accession GSE48145) [71]; scheduled for release 30 October 2013.

### Quantitative RT-PCR

RNA was prepared with TRIzol (Invitrogen) followed by ammonium acetate precipitation and DNAse treatment, quantified using NanoDrop ND-100. DNase-treated total RNA was reverse-transcribed with iScript cDNA synthesis (Bio-Rad Laboratories, Inc., Hercules, CA, USA.). Quantitative PCR was conducted with i*Taq* SYBR Green Supermix plus ROX (Bio-Rad) upon an ABI prism 7300 Sequence Detection System (Applied Biosystems, Life Technologies, Inc., Carlsbad, CA, USA.). Each estimate of mRNA was measured from four biological replicates, each with three technical replicates. Transcripts were normalized to *RPL32* mRNA control using the analytical method of 2^−ΔΔ*CT*^[62]. Primer sets are listed in (Additional file 6: Table S4).

## Abbreviations

CA: *Corpora allata*; CAKO: *Corpora allata* knockout; FbP1: Fat body protein 1; GCE: Germ-cell expressed bHLH-PAS; InR: Insulin-like receptor; JH: Juvenile hormone; JHA: Juvenile hormone analog; JHBIII: Bisepoxide JHIII; LSP1: Larval serum protein; MET: Methoprene-tolerant; MF: Methyl farnesoate; MSD/DS: Mass selective detector/detection software; NIPP1: Nuclear inhibitor protein phosphatase type 1; rpm: Revolutions per minute; WT: Wildtype.

## Competing interests

The authors declare that they have no competing interests.

## Authors' contributions

MT, RY and HB conceived and designed this study. RY and HB carried out the experiments. GA and AD measured JH titers from provided samples. MT analyzed the data and wrote the manuscript. All authors read and approved the final manuscript.

## Supplementary Material

Additional file 1: Table S1 Life table statistics. Click here for file

Additional file 2: Figure S1 Mortality rate plots for females in Figure 4. Click here for file

Additional file 3: Figure S2 Survival and mortality plots for CAKO males. Click here for file

Additional file 4: Table S2 Genes of group 1 and 2: responsive to reduced JH independent of reproductive genotype. Click here for file

Additional file 5: Table S3 Genes responsive to reduced JH in a single reproductive genotype. Click here for file

Additional file 6: Table S4 Primers for qPCR. Click here for file
