# Supplementary material for: Juvenile hormone regulation of Drosophila aging
Source: BMC Biol. 2013 Jul 17;11:85. doi: 10.1186/1741-7007-11-85 (PMC3726347; doi:10.1186/1741-7007-11-85)
Supplement: Additional file 1: Table S1 — Life table statistics. [file 1741-7007-11-85-S1.pdf]

Table S1

Figure	Genotype	diet or treatment	sex	Life expectancy (adult) median	LCL	UCL	N_0	Log-rank prob.(vs. CAKO)	Proportional Hazard relative risk (CAKO/WT)	
Fig 4A	CAKO (in w^{1118})	yeast 2%	female	44	44	46	279			
	NIPP1/w^{1118}	yeast 2%	female	30	30	30	122	< 0.001		
	w^{1118}/w^{1118}	yeast 2%	female	32	30	32	275	< 0.001		
	Aug21,GFP/w^{1118}	yeast 2%	female	32	30	32	225	< 0.001		
Fig 4B	CAKO (in w^{Dah})	yeast 2%	female	56	54	58	574			
	w^{Dah}	yeast 2%	female	48	46	48	478	< 0.001		
Fig 4C	CAKO	EtOH	female	54	52	56	522			
	wildtype	EtOH	female	42	42	44	507	< 0.001		
	CAKO	JHA	female	40	40	42	517	< 0.001		
	wildtype	JHA	female	40	38	40	532	< 0.001		
Fig 4D	CAKO, OvoD (in w^{Dah})	yeast 2%	female	48	46	50	574			
	OvoD (in w^{Dah})	yeast 2%	female	36	34	36	578	< 0.001		
Fig 5	CAKO (in w^{Dah})	yeast 1%	female	64	62	66	342		0.725	a
	CAKO (in w^{Dah})	yeast 2%	female	60	60	62	250		0.835	b
	CAKO (in w^{Dah})	yeast 4%	female	62	62	64	328		0.734	a
	CAKO (in w^{Dah})	yeast 8%	female	56	54	56	351		0.863	b
	CAKO (in w^{Dah})	yeast 16%	female	54	52	54	257		0.856	b
	w^{Dah}/w^{Dah}	yeast 1%	female	58	56	58	348	< 0.001		
	w^{Dah}/w^{Dah}	yeast 2%	female	58	56	58	326	< 0.001		
	w^{Dah}/w^{Dah}	yeast 4%	female	56	54	56	372	< 0.001		
	w^{Dah}/w^{Dah}	yeast 8%	female	52	52	54	330	< 0.001		
	w^{Dah}/w^{Dah}	yeast 16%	female	50	50	52	352	< 0.001		
Fig S2	CAKO (in w^{1118})	yeast 2%	male	36	34	38	431			
	w^{1118}/w^{1118}	yeast 2%	male	30	30	30	386	< 0.001		
	Aug21,GFP/w^{1118}	yeast 2%	male	28	28	30	420	< 0.001		
