# Supplementary figures and images for: Juvenile hormone regulation of Drosophila aging
Source: BMC Biol. 2013 Jul 17;11:85. doi: 10.1186/1741-7007-11-85 (PMC3726347; doi:10.1186/1741-7007-11-85)

Figure S1. Mortality rate plots corresponding to survival trials of text figure 4.

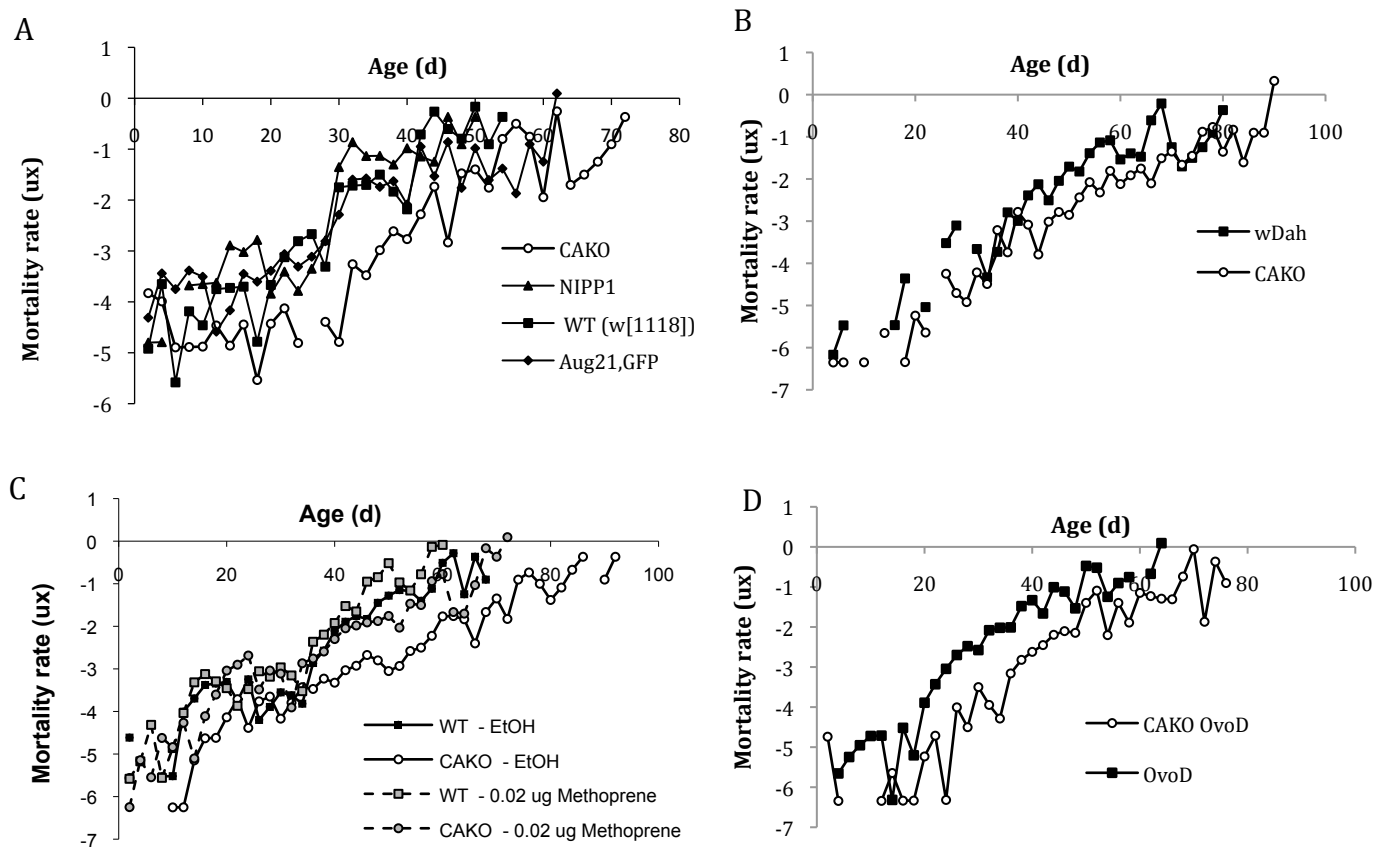

Supplement: Additional file 2: Figure S1 — Mortality rate plots for females in Figure 4. [file 1741-7007-11-85-S2.pdf]
