# Supplementary material for: Juvenile hormone regulation of Drosophila aging
Source: BMC Biol. 2013 Jul 17;11:85. doi: 10.1186/1741-7007-11-85 (PMC3726347; doi:10.1186/1741-7007-11-85)

Figure S2. Male life expectancy extended by CAKO, relative to controls *w1118* and *Aug21,GFP*/+. A) Survival. B) Mortality rate.

A)

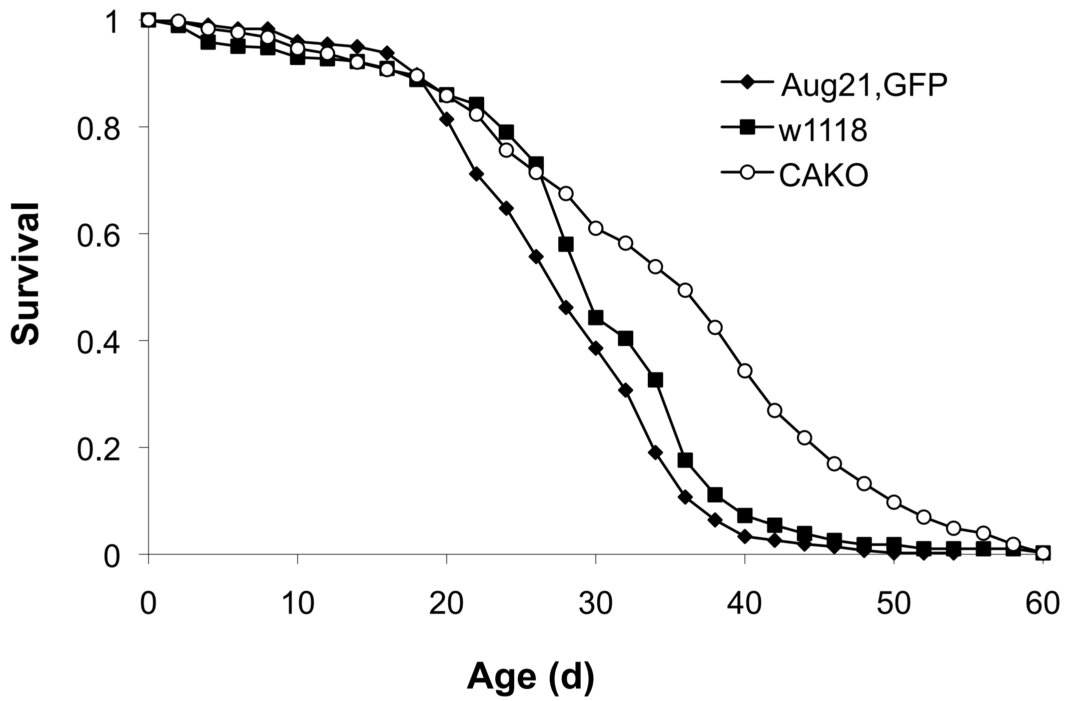

B)

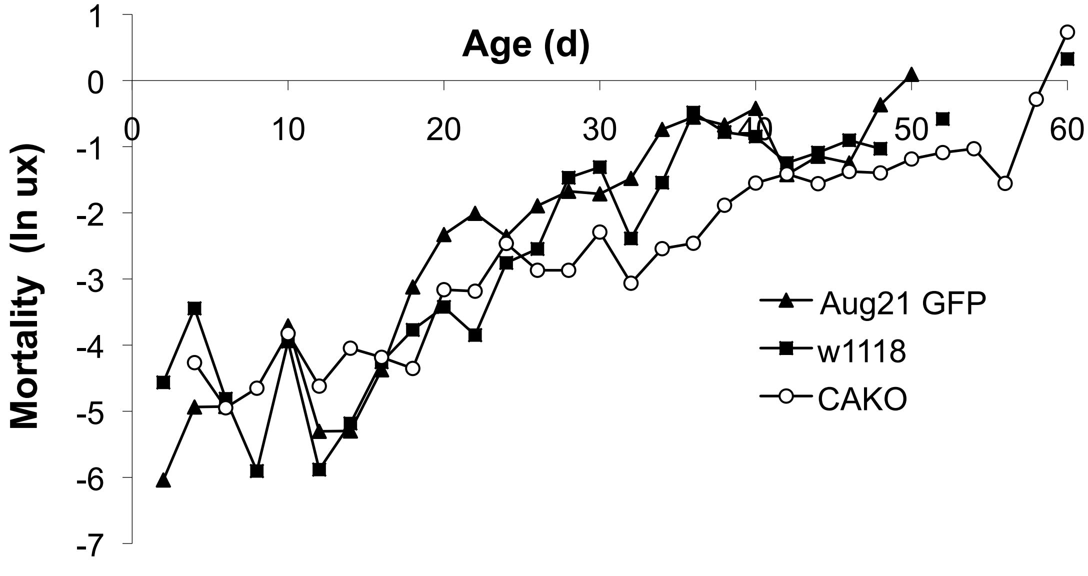

Supplement: Additional file 3: Figure S2 — Survival and mortality plots for CAKO males. [file 1741-7007-11-85-S3.pdf]
