# Supplementary material for: Juvenile hormone regulation of Drosophila aging
Source: BMC Biol. 2013 Jul 17;11:85. doi: 10.1186/1741-7007-11-85 (PMC3726347; doi:10.1186/1741-7007-11-85)
Supplement: Additional file 4: Table S2 — Genes of group 1 and 2: responsive to reduced JH independent of reproductive genotype. [file 1741-7007-11-85-S4.pdf]

Table S2

Group 1: Genes repressed by JH in both fertile and sterile females								
	**Normalized mRNA abundance**				**Fold change**		***p*-value**	
**Gene Symbol**	**wildtype**	**CAKO**	**OvoD**	**OvoD;CAKO**	**wildtype v. CAKO**	**OvoD v. OvoD;CAKO**	**wildtype v. CAKO**	**OvoD v. OvoD;CAKO**
CG1304	2.754	7.309	2.994	9.173	2.654	3.064	0.0001	0.0002
CG34040	4.677	11.286	10.287	12.569	2.413	1.222	0.0005	0.0032
CG2444	2.454	5.625	5.754	6.959	2.292	1.210	0.0007	0.0026
Obp99b	4.161	9.070	8.401	11.636	2.180	1.385	0.0009	0.0103
CG6012	2.861	6.140	4.419	8.099	2.146	1.833	0.0003	0.0032
CG31955	3.374	6.932	6.196	7.659	2.055	1.236	0.0014	0.0157
Sr-CIV	2.254	4.151	4.171	6.735	1.841	1.615	0.0016	0.0018
w	3.181	5.737	6.289	7.511	1.804	1.194	0.0086	0.0247
CG18302	3.521	6.194	5.740	8.115	1.759	1.414	0.0005	0.0009
CG15534	4.686	7.842	8.273	10.069	1.673	1.217	0.0057	0.0023
Cpr60D	2.761	4.613	5.566	6.368	1.671	1.144	0.0057	0.0337
Acp65Aa	3.275	5.339	4.795	7.087	1.631	1.478	0.0055	0.0017
Cyp309a1	5.274	8.585	9.143	10.700	1.628	1.170	0.0021	0.0019
CG33282	3.095	4.970	5.421	7.176	1.606	1.324	0.0390	0.0021
CR13656	3.673	5.667	5.865	7.970	1.543	1.359	0.0079	0.0039
CG10559	3.950	6.083	7.950	9.657	1.540	1.215	0.0003	0.0070
CG11893	4.172	6.311	5.898	8.180	1.513	1.387	0.0024	0.0148
CG12998	5.743	8.502	8.588	10.091	1.481	1.175	0.0028	0.0000
CG12256	3.361	4.895	5.488	6.939	1.456	1.264	0.0067	0.0070
Try29F	7.237	10.367	9.244	12.123	1.433	1.311	0.0029	0.0001
CG6385	3.661	5.129	4.831	6.395	1.401	1.324	0.0349	0.0082
CG15533	4.093	5.593	7.992	8.913	1.367	1.115	0.0115	0.0219
CG7135	4.235	5.559	5.461	7.283	1.312	1.334	0.0006	0.0012
Cyp313a1	7.730	10.140	9.334	10.947	1.312	1.173	0.0003	0.0002
CG1208	7.904	10.266	9.657	11.370	1.299	1.177	0.0014	0.0001
Ser6	7.536	9.725	8.831	10.848	1.291	1.228	0.0002	0.0017
yellow-b	5.457	7.023	7.829	8.634	1.287	1.103	0.0070	0.0129
DsimCG6640	8.669	10.866	11.732	12.966	1.253	1.105	0.0287	0.0316
CG31370	4.653	5.787	6.509	7.208	1.244	1.107	0.0049	0.0045
CG5724	8.874	10.847	11.483	12.650	1.222	1.102	0.0081	0.0004
Sodh-2	6.403	7.708	7.756	9.017	1.204	1.163	0.0095	0.0005
CG13856	4.481	5.363	5.475	7.115	1.197	1.300	0.0045	0.0012
Fuca	6.138	7.323	7.982	8.794	1.193	1.102	0.0055	0.0119
Cyp12d1-d	6.038	7.173	6.597	8.476	1.188	1.285	0.0087	0.0009
CG32982	3.615	4.278	4.164	5.054	1.183	1.214	0.0190	0.0164
sxe2	4.992	5.902	6.507	7.837	1.182	1.204	0.0142	0.0154
CG13177	4.988	5.890	6.288	6.990	1.181	1.112	0.0234	0.0461
Cpr66D	7.253	8.550	7.656	9.238	1.179	1.207	0.0319	0.0004
CG11668	2.722	3.201	3.547	4.114	1.176	1.160	0.0351	0.0258

Table S2

nmo	6.429	7.554	8.555	9.559	1.175	1.117	0.0194	0.0003
CG9993	5.436	6.378	6.766	8.283	1.173	1.224	0.0483	0.0037
CG15553	2.501	2.914	2.860	5.613	1.165	1.963	0.0006	0.0071
obst-E	6.537	7.616	7.873	8.971	1.165	1.139	0.0040	0.0010
CG15434	7.012	8.137	8.000	8.860	1.160	1.107	0.0006	0.0108
CG9400	5.671	6.568	6.573	7.339	1.158	1.116	0.0289	0.0413
CG2064	7.668	8.786	9.421	10.577	1.146	1.123	0.0211	0.0043
CG14607	3.329	3.805	4.326	4.897	1.143	1.132	0.0025	0.0005
CG33256	6.257	7.150	7.554	8.341	1.143	1.104	0.0137	0.0330
CG15617	4.932	5.540	5.936	6.923	1.123	1.166	0.0368	0.0426
CG32726	5.965	6.657	7.273	8.131	1.116	1.118	0.0010	0.0042
CG14934	9.899	10.988	9.888	11.954	1.110	1.209	0.0211	0.0008
lush	6.105	6.742	7.393	8.152	1.104	1.103	0.0016	0.0304
Group 2: Genes induced by JH in both fertile and sterile females								
	**Normalized mRNA abundance**				**Fold change**		***p*-value**	
**Gene Symbol**	**wildtype**	**CAKO**	**OvoD**	**OvoD;CAKO**	**wildtype v. CAKO**	**OvoD v. OvoD;CAKO**	**wildtype v. CAKO**	**OvoD v. OvoD;CAKO**
Jon25Bii	9.295	3.951	11.762	7.743	2.353	1.519	0.0025	0.0500
CG31259	5.539	2.368	6.063	3.879	2.339	1.563	0.0114	0.0210
CG31775	7.987	4.833	7.539	4.389	1.653	1.718	0.0022	0.0000
Jon25Bi	8.296	5.118	11.372	8.434	1.621	1.348	0.0073	0.0351
CG5246	7.101	4.418	10.025	7.077	1.607	1.416	0.0106	0.0045
CG2772	5.368	3.533	8.806	5.377	1.520	1.638	0.0205	0.0000
jhamt	4.654	3.129	6.647	4.303	1.488	1.545	0.0110	0.0115
CG33109	10.751	7.444	12.612	10.546	1.444	1.196	0.0044	0.0027
CG3106	7.672	5.561	8.161	7.382	1.380	1.105	0.0012	0.0019
CG17239	8.261	6.019	10.494	8.763	1.372	1.198	0.0293	0.0125
CG10182	4.495	3.277	6.857	5.449	1.372	1.259	0.0234	0.0296
CG31041	11.327	8.383	11.684	9.271	1.351	1.260	0.0000	0.0060
CG12970	3.258	2.471	4.355	2.997	1.318	1.453	0.0356	0.0031
CG6839	8.221	6.446	9.487	7.585	1.275	1.251	0.0171	0.0001
CG32207	6.947	5.466	4.907	2.329	1.271	2.107	0.0208	0.0072
CG13947	7.752	6.174	10.322	7.385	1.256	1.398	0.0242	0.0000
fit	11.158	8.942	14.224	12.589	1.248	1.130	0.0017	0.0414
CG7298	11.752	9.428	11.765	10.638	1.246	1.106	0.0115	0.0362
ana	8.179	6.603	9.915	8.765	1.239	1.131	0.0010	0.0015
Vm26Ab	13.361	10.994	4.685	3.209	1.215	1.460	0.0024	0.0306
CG17012	9.320	7.699	11.876	10.156	1.211	1.169	0.0042	0.0009
CG7567	7.678	6.346	10.387	7.940	1.210	1.308	0.0388	0.0307
CG2177	8.083	6.681	8.379	7.082	1.210	1.183	0.0060	0.0006
CG7631	8.155	6.744	10.591	9.187	1.209	1.153	0.0290	0.0014

Table S2

Pde1c	5.032	4.253	6.288	5.370	1.183	1.171	0.0007	0.0011
CG2022	7.386	6.274	9.039	7.561	1.177	1.196	0.0247	0.0020
CG11912	9.594	8.167	11.179	10.069	1.175	1.110	0.0108	0.0053
CG4822	9.376	7.989	10.468	9.305	1.174	1.125	0.0000	0.0077
CG34005	7.177	6.201	8.742	7.717	1.157	1.133	0.0278	0.0267
CG31759	5.567	4.825	4.611	3.890	1.154	1.185	0.0139	0.0023
phr6-4	7.952	6.934	4.093	3.029	1.147	1.351	0.0007	0.0356
Drep-2	3.613	3.179	2.543	2.262	1.137	1.124	0.0399	0.0668
CG13912	9.291	8.177	9.953	8.991	1.136	1.107	0.0033	0.0021
Cyp4ac3	6.945	6.116	8.201	7.307	1.136	1.122	0.0047	0.0019
SCAP	9.752	8.614	9.538	8.361	1.132	1.141	0.0067	0.0225
Invadolysin	7.103	6.324	7.819	6.502	1.123	1.203	0.0382	0.0072
CG9616	2.646	2.373	7.507	3.434	1.115	2.186	0.0342	0.0087
CG33120	7.558	6.820	9.928	8.830	1.108	1.124	0.0269	0.0207
nimC1	6.181	5.594	9.066	8.013	1.105	1.131	0.0008	0.0018
Cyp4ad1	7.583	6.867	8.952	8.097	1.104	1.106	0.0195	0.0039
CG30438	5.213	4.723	8.633	6.153	1.104	1.403	0.0275	0.0004
caps	2.727	2.472	3.304	2.803	1.103	1.178	0.0230	0.0077
