# Supplementary material for: Juvenile hormone regulation of Drosophila aging
Source: BMC Biol. 2013 Jul 17;11:85. doi: 10.1186/1741-7007-11-85 (PMC3726347; doi:10.1186/1741-7007-11-85)
Supplement: Additional file 5: Table S3 — Genes responsive to reduced JH in a single reproductive genotype. [file 1741-7007-11-85-S5.pdf]

Table S3

Genes repressed by JH in fertile but not Ovo^{D1} (sterile) females								
	**Normalized mRNA abundance**				**Fold change**		***p*-value**	
**Gene Symbol**	**wildtype**	**CAKO**	**OvoD**	**OvoD;CAKO**	**wildtype v. CAKO**	**OvoD v. OvoD;CAKO**	**wildtype v. CAKO**	**OvoD v. OvoD;CAKO**
Lsp2	3.8417	8.1996	14.0669	13.0575	2.134	1.077	0.003	0.038
CG4500	3.0822	5.7567	7.9526	6.7290	1.868	1.182	0.002	0.048
CG1139	4.6726	8.0554	8.7219	9.4754	1.724	0.920	0.001	0.006
Lsp1beta	6.0542	10.0328	11.5188	12.5909	1.657	0.915	0.000	0.030
CG17751	4.1614	6.6069	8.9193	9.1886	1.588	0.971	0.002	0.243
CG31664	2.8624	4.4820	6.6404	6.9513	1.566	0.955	0.000	0.248
CG6271	7.0599	10.0174	11.3693	11.4215	1.419	0.995	0.001	0.698
CG33469	3.5839	5.0365	7.0574	7.0983	1.405	0.994	0.003	0.875
CS-2	4.8528	6.7900	8.1792	8.1536	1.399	1.003	0.004	0.786
CG6283	7.8058	10.7761	11.3075	11.9390	1.381	0.947	0.000	0.000
CG14694	5.1702	6.9212	8.3221	8.6406	1.339	0.963	0.000	0.040
CG16898	8.8292	11.5098	11.6986	12.7091	1.304	0.920	0.002	0.003
CG8093	7.6438	9.8834	11.6222	11.8753	1.293	0.979	0.003	0.054
CG41284	3.5319	4.5663	3.0556	3.0616	1.293	0.998	0.000	0.970
CG13636	6.8450	8.7637	7.0547	7.2847	1.280	0.968	0.003	0.352
CG13609	4.8785	6.1990	2.6370	2.6195	1.271	1.007	0.004	0.777
CG12721	5.4641	6.9205	4.4677	4.3612	1.267	1.024	0.003	0.474
CG32444	10.3768	13.1014	13.3465	14.1227	1.263	0.945	0.003	0.009
Sodh-1	9.3633	11.7371	12.7832	13.2839	1.254	0.962	0.002	0.099
CG3301	5.4982	6.8808	8.4150	8.7964	1.251	0.957	0.001	0.057
CG3699	8.7384	10.9245	11.9764	12.9411	1.250	0.925	0.005	0.001
CG10621	7.1141	8.8576	11.0741	10.7155	1.245	1.033	0.000	0.048
CG15865	4.4817	5.5470	8.4472	8.5040	1.238	0.993	0.001	0.647
CG12268	4.7509	5.8616	7.2248	7.2995	1.234	0.990	0.002	0.722
yellow-f	3.1782	3.8892	5.5938	4.9601	1.224	1.128	0.060	0.047
Gen	4.1016	5.0060	2.9680	2.5437	1.221	1.167	0.033	0.009
smp-30	9.7261	11.8592	12.4079	12.9624	1.219	0.957	0.001	0.021
CG9981	2.2419	2.7333	4.1490	3.0876	1.219	1.344	0.002	0.063
CG4741	4.6934	5.7114	6.3333	6.9163	1.217	0.916	0.000	0.111
CG9664	5.3057	6.4194	8.5168	8.2521	1.210	1.032	0.002	0.101
yellow-c	7.3723	8.9066	9.5695	10.2160	1.208	0.937	0.001	0.029
CG1889	5.9699	7.2095	8.7684	8.9237	1.208	0.983	0.005	0.263
CG17189	6.6169	7.9552	9.0568	9.3892	1.202	0.965	0.004	0.121
CG10050	4.7688	5.7225	2.5769	2.7575	1.200	0.935	0.001	0.457
CG15120	5.6832	6.7953	8.7304	8.5327	1.196	1.023	0.001	0.518
Obp56a	7.9531	9.4861	10.3565	11.2691	1.193	0.919	0.002	0.009
CG3032	2.5026	2.9839	2.4145	2.4118	1.192	1.001	0.001	0.260
CG15408	6.7946	8.0975	10.0339	10.3216	1.192	0.972	0.002	0.084
CG16965	6.1652	7.2895	9.4866	10.0461	1.182	0.944	0.000	0.040
CG1773	6.5629	7.7404	9.4639	9.4220	1.179	1.004	0.004	0.773

Table S3

CG7968	8.2323	9.7085	11.1907	11.6945	1.179	0.957	0.002	0.001
Cyp309a2	6.4511	7.5966	9.0112	9.5320	1.178	0.945	0.002	0.169
CG30427	5.9985	7.0617	8.0328	8.7535	1.177	0.918	0.002	0.001
CG9510	9.2576	10.8466	11.9666	12.3912	1.172	0.966	0.000	0.070
CG5397	7.4309	8.7004	9.5170	10.2170	1.171	0.931	0.004	0.006
CG6830	8.7720	10.2612	11.4902	12.1184	1.170	0.948	0.002	0.001
CG5895	5.8461	6.8193	7.7903	7.9699	1.166	0.977	0.001	0.321
CG31189	5.0149	5.8443	6.9074	7.1639	1.165	0.964	0.002	0.071
CG2082	7.9643	9.2788	9.4766	10.2046	1.165	0.929	0.004	0.017
Cyp28d1	10.6823	12.4441	13.0908	13.3571	1.165	0.980	0.005	0.060
CG6484	9.9022	11.5138	11.9089	12.4934	1.163	0.953	0.001	0.003
Ugt86Dc	6.5329	7.5922	9.7141	9.1279	1.162	1.064	0.000	0.033
CG11852	7.5561	8.7626	9.2988	10.1468	1.160	0.916	0.001	0.002
CG11878	10.1004	11.7057	12.1855	12.8238	1.159	0.950	0.001	0.002
CG10516	8.0425	9.3094	10.8189	10.5902	1.158	1.022	0.002	0.031
CG30345	8.7157	10.0659	10.7061	11.0241	1.155	0.971	0.000	0.076
CG10131	5.1242	5.9014	7.8909	7.8183	1.152	1.009	0.001	0.594
CG9512	10.7596	12.3714	12.8745	13.5874	1.150	0.948	0.000	0.005
dro4	10.5366	12.1115	12.4545	13.0101	1.149	0.957	0.002	0.019
CG15353	8.2467	9.4779	10.2058	10.9888	1.149	0.929	0.001	0.007
GRHRII	6.3600	7.2948	8.7604	8.7712	1.147	0.999	0.003	0.916
Lectin-galC1	10.9149	12.5066	12.6434	13.3186	1.146	0.949	0.000	0.005
CG6435	6.4569	7.3930	8.7528	8.8265	1.145	0.992	0.004	0.463
CG11919	8.0188	9.1698	10.5763	11.1259	1.144	0.951	0.003	0.047
yellow-f2	6.8435	7.8223	9.1131	9.3305	1.143	0.977	0.001	0.165
CG14259	7.8851	9.0010	10.0002	10.5582	1.142	0.947	0.002	0.007
wdb	5.1177	5.8314	3.1780	2.8058	1.139	1.133	0.127	0.028
ninaE	8.8142	9.9996	10.4596	11.3131	1.134	0.925	0.004	0.051
CG18522	10.0764	11.4311	11.8698	12.3054	1.134	0.965	0.000	0.005
CG9119	9.6309	10.9001	11.6040	12.3076	1.132	0.943	0.002	0.001
CG14375	9.2915	10.5127	12.1635	12.0181	1.131	1.012	0.000	0.067
Faa	7.0885	8.0137	9.0000	9.4109	1.131	0.956	0.001	0.039
CG3609	10.2044	11.5189	12.0613	12.8355	1.129	0.940	0.005	0.001
CG15358	7.3233	8.2661	9.4244	10.0357	1.129	0.939	0.002	0.038
CG9497	7.4026	8.3478	9.0532	9.7346	1.128	0.930	0.001	0.026
CG8012	9.2102	10.3851	11.3984	11.4129	1.128	0.999	0.000	0.832
Tsf1	11.9143	13.4249	14.2733	14.6517	1.127	0.974	0.001	0.008
Ugt36Bc	8.9225	10.0444	11.0196	11.5367	1.126	0.955	0.003	0.003
Ahcy89E	7.0703	7.9553	8.8639	9.5162	1.125	0.931	0.001	0.003
CG9312	7.7316	8.6948	9.8633	10.1640	1.125	0.970	0.004	0.018
a5	5.0673	5.6904	6.1427	6.4028	1.123	0.959	0.003	0.603
Mgstl	8.1005	9.0913	10.2969	10.5594	1.122	0.975	0.001	0.115

Table S3

yellow-e	5.6490	6.3349	8.1462	8.2475	1.121	0.988	0.003	0.261
CG7763	2.1475	2.4057	4.4046	2.4649	1.120	1.787	0.419	0.033
CG31313	11.5012	12.8839	13.2250	13.8711	1.120	0.953	0.005	0.000
Cyp12a5	8.6487	9.6824	10.6668	11.4379	1.120	0.933	0.003	0.002
CG4377	11.3807	12.7405	13.2933	13.8165	1.119	0.962	0.003	0.001
Mur18B	12.0073	13.4364	13.9511	14.3177	1.119	0.974	0.001	0.010
CG33514	8.6110	9.6169	10.7677	11.2210	1.117	0.960	0.000	0.005
CG12896	9.9833	11.1319	12.5238	12.2635	1.115	1.021	0.002	0.113
CG18547	8.8894	9.9088	10.6807	11.0641	1.115	0.965	0.000	0.006
CG13139	6.4867	7.2277	8.0424	8.7221	1.114	0.922	0.002	0.027
CG6834	7.1613	7.9733	9.1846	9.6632	1.113	0.950	0.002	0.026
CG15406	8.0435	8.9531	10.0199	10.3282	1.113	0.970	0.003	0.009
Cyp4ae1	6.0433	6.7225	8.3038	8.4510	1.112	0.983	0.002	0.204
CG1946	5.7676	6.4153	8.5039	7.5907	1.112	1.120	0.104	0.023
CG3270	8.7354	9.7049	10.5139	11.4425	1.111	0.919	0.000	0.002
CG9903	5.0074	5.5621	7.5930	7.5390	1.111	1.007	0.001	0.568
CG31199	8.1250	9.0218	10.2198	10.6748	1.110	0.957	0.003	0.016
CG7953	11.2798	12.5017	13.5211	13.8313	1.108	0.978	0.004	0.135
CG5160	2.3847	2.6423	3.0273	2.8592	1.108	1.059	0.001	0.629
CG13707	5.8439	6.4670	7.3943	7.7673	1.107	0.952	0.002	0.015
CG3857	7.4542	8.2483	11.1531	9.5141	1.107	1.172	0.006	0.001
Buffy	7.0413	7.7899	8.4713	8.7087	1.106	0.973	0.003	0.186
CG16997	9.6163	10.6039	11.3976	12.1191	1.103	0.940	0.002	0.002
regucalcin	8.5778	9.4588	11.1008	10.9685	1.103	1.012	0.003	0.348
st	7.1836	7.9179	9.2533	9.6086	1.102	0.963	0.003	0.013
Tsp42El	8.0873	8.9132	10.0292	10.2669	1.102	0.977	0.003	0.014
CG5577	8.1718	9.0062	10.1468	10.3889	1.102	0.977	0.001	0.073
CG30360	12.2508	13.4800	13.8189	14.1966	1.100	0.973	0.003	0.026
CG33178	8.7045	9.5760	10.3667	10.7736	1.100	0.962	0.000	0.001
Genes induced by JH in fertile but not Ovo^{D1} (sterile) females								
	**Normalized mRNA abundance**				**Fold change**		***p*-value**	
**Gene Symbol**	**wildtype**	**CAKO**	**OvoD**	**OvoD;CAKO**	**wildtype v. CAKO**	**OvoD v. OvoD;CAKO**	**wildtype v. CAKO**	**OvoD v. OvoD;CAKO**
Osi6	10.087	2.134	2.134	2.134	4.727	1.000	0.004	NA
CG40298	5.939	2.167	2.412	2.231	2.741	1.081	0.000	0.113
Osi15	7.546	2.907	2.134	2.134	2.596	1.000	0.012	NA
CG31926	5.611	2.426	2.147	2.147	2.313	1.000	0.026	0.623
TwdlN	7.111	3.092	2.155	2.157	2.300	0.999	0.034	0.257
Cpr65Ax1	6.968	3.106	2.134	2.134	2.243	1.000	0.012	0.423
CG6704	6.252	2.815	2.164	2.227	2.221	0.972	0.001	0.212
CG7465	4.604	2.277	2.372	2.280	2.022	1.040	0.000	0.437

Table S3

ImpE2	4.192	2.134	2.134	2.134	1.965	1.000	0.045	NA	
Cpr47Eg	4.281	2.225	2.211	2.246	1.924	0.984	0.000	0.038
CG10953	4.416	2.311	2.329	2.308	1.911	1.009	0.018	0.449
CG17192	8.392	4.416	10.047	9.143	1.900	1.099	0.005	0.204
CG17290	6.527	3.572	2.135	2.135	1.827	1.000	0.045	0.438
CG13679	3.890	2.186	2.157	2.157	1.780	1.000	0.008	0.876
CG31813	7.580	4.341	3.241	3.244	1.746	0.999	0.035	0.523
Obp19c	8.715	5.235	3.101	3.073	1.665	1.009	0.048	0.785
CG11741	5.419	3.262	5.171	5.446	1.661	0.949	0.046	0.542
CG31928	5.822	3.524	2.242	2.621	1.652	0.856	0.015	0.064
CG8147	10.087	6.124	4.800	5.054	1.647	0.950	0.017	0.732
CG14187	6.160	3.796	2.463	2.325	1.623	1.059	0.039	0.360
CG42335	3.885	2.426	5.502	5.964	1.602	0.923	0.044	0.320
CG8960	3.388	2.140	2.139	2.138	1.583	1.001	0.023	0.521
CG13992	5.245	3.384	3.147	3.228	1.550	0.975	0.017	0.143
CG3323	3.276	2.134	2.134	2.134	1.535	1.000	0.000	NA
l(2)03659	6.038	4.008	3.076	3.005	1.507	1.024	0.002	0.659
CG11873	4.249	2.856	2.240	2.240	1.488	1.000	0.016	0.663
CG12517	5.092	3.453	3.093	3.135	1.475	0.987	0.030	0.595
CG9505	4.053	2.761	2.772	2.886	1.468	0.960	0.012	0.515
CG5326	5.663	3.934	3.729	3.731	1.440	0.999	0.033	0.993
vanin-like	8.906	6.232	8.748	8.336	1.429	1.049	0.007	0.037
se	5.668	3.986	5.397	5.510	1.422	0.979	0.012	0.701
CG8083	7.904	5.685	8.106	8.165	1.390	0.993	0.026	0.825
CG4115	6.251	4.584	5.730	6.335	1.364	0.904	0.041	0.003
CG5150	10.633	7.825	10.870	10.299	1.359	1.055	0.001	0.056
TwdlG	5.853	4.407	4.756	4.480	1.328	1.062	0.013	0.452
CG7912	4.409	3.325	4.356	4.116	1.326	1.058	0.017	0.230
Tequila	5.409	4.088	6.067	5.963	1.323	1.017	0.002	0.581
CG3348	10.896	8.335	10.322	10.289	1.307	1.003	0.008	0.939
CG8785	9.458	7.237	9.780	9.123	1.307	1.072	0.003	0.038
Fcp26Aa	8.074	6.197	3.367	3.166	1.303	1.064	0.012	0.459
br	4.964	3.839	3.456	3.304	1.293	1.046	0.017	0.634
CG10592	11.533	8.937	11.872	11.001	1.290	1.079	0.004	0.008
pip	2.910	2.269	2.216	2.225	1.283	0.996	0.022	0.864
CG18179	5.171	4.041	8.412	8.050	1.280	1.045	0.007	0.639
LKR	5.617	4.476	6.461	6.205	1.255	1.041	0.039	0.438
CG10725	9.620	7.788	9.900	9.217	1.235	1.074	0.024	0.030
Kr-h1	9.414	7.642	9.206	9.104	1.232	1.011	0.025	0.416
CG30043	5.094	4.172	5.808	6.507	1.221	0.893	0.015	0.078
CG11093	3.615	2.984	4.347	4.013	1.211	1.083	0.015	0.102
CG32773	2.709	2.240	2.235	2.235	1.210	1.000	0.001	0.387
CG33097	6.351	5.255	5.432	5.198	1.209	1.045	0.042	0.348
Odc1	10.358	8.578	11.024	10.142	1.208	1.087	0.001	0.005

Table S3

CG15829	9.433	7.831	12.129	11.030	1.205	1.100	0.007	0.076
CG31159	4.290	3.564	3.262	3.147	1.204	1.037	0.021	0.518
CG3290	11.976	9.951	11.620	11.264	1.204	1.032	0.005	0.076
Fcp3C	9.623	8.030	3.111	3.077	1.198	1.011	0.049	0.874
Cht5	3.438	2.877	4.849	4.605	1.195	1.053	0.043	0.676
Lcp65Ag2	9.114	7.628	5.171	7.221	1.195	0.716	0.207	0.030
CG5550	9.199	7.701	10.632	9.843	1.194	1.080	0.016	0.039
Ilp6	6.936	5.810	6.527	6.356	1.194	1.027	0.001	0.296
SP71	3.233	2.708	2.790	2.786	1.194	1.001	0.031	0.273
Vm32E	11.885	9.967	2.363	2.388	1.193	0.990	0.031	0.756
CG18594	13.423	11.261	14.550	13.745	1.192	1.059	0.003	0.019
Hsp70Aa	4.919	4.142	4.662	5.817	1.188	0.802	0.005	0.001
Cpr64Ad	4.157	3.517	3.442	3.442	1.182	1.000	0.004	0.982
CG4098	8.679	7.347	7.345	6.753	1.181	1.088	0.005	0.066
CG18539	4.234	3.592	5.326	4.971	1.179	1.071	0.009	0.498
tadr	4.162	3.544	4.987	5.211	1.174	0.957	0.046	0.094
CG14798	5.945	5.071	3.185	3.538	1.172	0.900	0.028	0.231
CG5767	10.562	9.060	12.026	11.055	1.166	1.088	0.010	0.003
CG15044	7.162	6.173	9.508	8.771	1.160	1.084	0.019	0.034
Tollo	9.460	8.178	8.152	7.868	1.157	1.036	0.002	0.070
CG6870	10.021	8.663	10.563	10.184	1.157	1.037	0.001	0.075
CG40485	6.623	5.744	6.668	6.820	1.153	0.978	0.000	0.264
Cap-H2	5.029	4.364	4.077	4.128	1.152	0.988	0.020	0.769
mus308	5.502	4.777	4.248	4.179	1.152	1.017	0.036	0.719
CG32284	6.997	6.082	6.126	7.375	1.151	0.831	0.294	0.019
CG16743	8.908	7.745	10.738	9.929	1.150	1.081	0.003	0.037
CG34253	5.382	4.693	6.594	6.258	1.147	1.054	0.008	0.143
CG34267	4.750	4.143	4.757	4.658	1.147	1.021	0.008	0.286
CG3819	11.374	9.969	11.327	11.249	1.141	1.007	0.040	0.463
dsx	8.301	7.285	9.692	9.259	1.140	1.047	0.021	0.006
Epac	7.214	6.340	7.780	7.601	1.138	1.024	0.030	0.364
wbl	7.257	6.386	7.264	7.353	1.136	0.988	0.008	0.602
CG8745	11.699	10.309	12.200	12.126	1.135	1.006	0.006	0.461
CG33173	10.237	9.060	11.497	10.724	1.130	1.072	0.029	0.017
CG6106	6.388	5.655	4.886	5.259	1.130	0.929	0.001	0.220
CG2292	3.831	3.397	3.582	3.408	1.128	1.051	0.031	0.484
Ten-a	6.961	6.172	7.011	7.140	1.128	0.982	0.040	0.219
ap	5.663	5.023	7.157	6.551	1.127	1.093	0.036	0.150
Mip	6.862	6.088	7.123	7.478	1.127	0.952	0.002	0.017
jing	5.583	4.975	4.061	3.980	1.122	1.020	0.005	0.529
Ela	8.392	7.495	9.967	9.086	1.120	1.097	0.026	0.002
Mt2	3.088	2.762	2.310	2.303	1.118	1.003	0.041	0.927
CG12026	8.571	7.668	6.354	6.191	1.118	1.026	0.018	0.368
CG13324	10.625	9.505	11.774	11.104	1.118	1.060	0.047	0.074

Table S3

ckd	3.699	3.314	4.362	4.639	1.116	0.940	0.012	0.448
Eip75B	7.393	6.632	8.047	7.897	1.115	1.019	0.036	0.236
S1P	8.111	7.280	6.734	6.715	1.114	1.003	0.006	0.865
Dot	6.507	5.842	7.556	7.274	1.114	1.039	0.026	0.110
CG11911	13.707	12.313	14.054	13.295	1.113	1.057	0.002	0.001
CG16857	6.375	5.730	6.734	6.621	1.113	1.017	0.000	0.397
Cyp6d2	8.639	7.766	8.116	8.353	1.112	0.972	0.001	0.165
CG14949	8.749	7.867	9.643	9.032	1.112	1.068	0.033	0.016
CG15546	5.329	4.792	6.204	6.755	1.112	0.918	0.029	0.042
CG10508	3.613	3.250	4.107	4.921	1.111	0.834	0.128	0.033
CG4259	8.252	7.426	9.715	9.261	1.111	1.049	0.000	0.083
CG9451	9.532	8.580	9.598	9.579	1.111	1.002	0.007	0.802
CG7367	3.301	2.975	3.527	3.559	1.110	0.991	0.040	0.905
Ag5r2	10.691	9.634	11.599	10.946	1.110	1.060	0.013	0.020
CG8303	8.655	7.810	6.468	6.231	1.108	1.038	0.016	0.139
argos	5.340	4.820	5.520	5.444	1.108	1.014	0.017	0.828
CG13299	10.701	9.673	8.163	9.050	1.106	0.902	0.043	0.008
NetA	5.668	5.124	6.593	6.396	1.106	1.031	0.024	0.311
CG34123	5.495	4.968	6.038	5.556	1.106	1.087	0.016	0.106
CG31054	6.505	5.884	4.254	4.006	1.106	1.062	0.017	0.256
CG11671	11.411	10.323	12.286	11.987	1.105	1.025	0.012	0.048
CG33090	9.189	8.316	8.221	8.016	1.105	1.026	0.007	0.285
CG2003	6.919	6.268	6.821	6.663	1.104	1.024	0.009	0.443
CG30046	5.415	4.906	5.663	5.571	1.104	1.017	0.013	0.424
CG14410	6.541	5.927	3.317	3.552	1.104	0.934	0.024	0.544
RpL3	9.143	8.285	8.087	7.967	1.103	1.015	0.015	0.336
lola	6.295	5.705	6.346	6.239	1.103	1.017	0.024	0.264
CG16791	6.696	6.070	7.830	7.714	1.103	1.015	0.031	0.474
CG34348	7.429	6.736	5.199	5.161	1.103	1.007	0.043	0.801
CG30440	9.421	8.552	7.404	7.408	1.102	0.999	0.001	0.984
CG8773	9.159	8.315	10.703	10.233	1.102	1.046	0.026	0.005
Eip71CD	10.151	9.218	9.334	9.758	1.101	0.957	0.047	0.013
CG5794	7.635	6.935	6.961	6.923	1.101	1.006	0.000	0.680
CG4842	7.851	7.135	6.926	6.869	1.100	1.008	0.009	0.691
Idgf1	10.105	9.184	11.036	10.588	1.100	1.042	0.004	0.002
CG31233	11.541	10.491	12.427	12.013	1.100	1.034	0.007	0.003
Genes repressed by JH in Ovo^{D1} (sterile) but not in fertile females								
	**Normalized mRNA abundance**				**Fold change**		***p*-value**	
**Gene Symbol**	**wildtype**	**CAKO**	**OvoD**	**OvoD;CAKO**	**wildtype v. CAKO**	**OvoD v. OvoD;CAKO**	**wildtype v. CAKO**	**OvoD v. OvoD;CAKO**
CG7768	2.155	2.297	2.550	4.010	1.066	1.572	0.028	0.014
Side	2.906	2.848	2.822	4.313	0.980	1.528	0.636	0.018
CG14642	2.963	2.909	2.983	4.555	0.982	1.527	0.455	0.002

Table S3

CG8483	3.152	3.263	3.164	4.727	1.035	1.494	0.256	0.000
Mlp60A	2.538	2.610	2.798	4.074	1.028	1.456	0.593	0.003
Lcp65Ag2	9.114	7.628	5.171	7.221	0.837	1.397	0.207	0.030
CG4927	2.431	2.587	2.584	3.608	1.064	1.396	0.017	0.038
Cyp6a17	9.110	9.629	7.665	10.582	1.057	1.381	0.016	0.018
skpB	3.008	3.200	3.428	4.532	1.064	1.322	0.308	0.014
CG33296	3.577	3.697	4.399	5.786	1.034	1.315	0.830	0.004
CG2650	2.153	2.194	5.826	7.552	1.019	1.296	0.321	0.023
mthl8	3.665	3.979	3.180	4.118	1.086	1.295	0.201	0.044
sev	2.136	2.147	2.248	2.910	1.005	1.294	0.269	0.005
Peritrophin-A	2.399	2.413	2.742	3.542	1.006	1.292	0.891	0.003
Cpr97Eb	3.392	3.504	3.613	4.664	1.033	1.291	0.360	0.002
CG15822	4.983	5.238	3.859	4.927	1.051	1.277	0.189	0.001
CG15711	2.602	2.741	3.176	4.049	1.053	1.275	0.117	0.039
CG32271	3.266	3.337	3.737	4.745	1.022	1.270	0.518	0.014
CG7810	6.617	6.576	4.791	6.027	0.994	1.258	0.713	0.025
eys	2.486	2.497	3.087	3.883	1.004	1.258	0.813	0.048
Hsp70Aa	4.919	4.142	4.662	5.817	0.842	1.248	0.005	0.001
CG32225	3.777	4.028	4.289	5.330	1.067	1.243	0.158	0.047
CG7201	3.990	3.907	4.167	5.144	0.979	1.235	0.482	0.006
CG3588	3.376	3.520	3.939	4.838	1.043	1.228	0.361	0.019
MED27	7.611	7.683	4.452	5.424	1.009	1.218	0.253	0.049
5-HT7	2.173	2.210	2.228	2.709	1.017	1.216	0.274	0.004
CG11839	8.518	8.406	5.237	6.307	0.987	1.204	0.532	0.045
Meics	7.751	7.915	5.242	6.311	1.021	1.204	0.146	0.012
CG32284	6.997	6.082	6.126	7.375	0.869	1.204	0.294	0.019
CG4537	4.567	4.227	3.211	3.863	0.926	1.203	0.343	0.009
CG6083	3.700	3.873	4.153	4.978	1.047	1.199	0.330	0.004
CG10508	3.613	3.250	4.107	4.921	0.900	1.198	0.128	0.033
CG6151	4.060	4.464	2.339	2.795	1.100	1.195	0.545	0.026
FucTA	3.317	3.348	3.672	4.382	1.009	1.194	0.900	0.048
CG11407	6.687	7.110	7.597	9.048	1.063	1.191	0.170	0.020
CG13101	6.957	7.537	6.920	8.175	1.083	1.181	0.008	0.008
Obp83g	3.039	2.930	3.406	4.021	0.964	1.181	0.763	0.024
CG18136	4.471	4.593	4.988	5.867	1.027	1.176	0.161	0.021
CG9279	2.826	2.879	2.903	3.409	1.019	1.174	0.295	0.034
CG31928	5.822	3.524	2.242	2.621	0.605	1.169	0.015	0.064
ect	4.820	5.057	5.431	6.331	1.049	1.166	0.362	0.041
rdgA	2.278	2.343	2.362	2.750	1.029	1.164	0.234	0.011
CG13188	4.773	5.115	4.997	5.819	1.072	1.164	0.014	0.001
bip1	9.659	9.507	6.168	7.173	0.984	1.163	0.195	0.001
CG4788	8.056	8.284	4.456	5.173	1.028	1.161	0.124	0.023
CG9272	7.891	8.203	5.766	6.692	1.039	1.161	0.043	0.001

Table S3

CG4726	4.986	5.026	5.414	6.264	1.008	1.157	0.816	0.018
SMC2	7.218	7.182	3.831	4.431	0.995	1.156	0.674	0.036
CG2837	3.521	3.678	3.788	4.380	1.045	1.156	0.240	0.044
SRm160	5.753	5.551	3.595	4.140	0.965	1.152	0.184	0.022
CG13001	6.454	6.825	4.204	4.819	1.057	1.146	0.208	0.035
Dfd	3.268	3.575	4.418	5.057	1.094	1.145	0.264	0.031
CG3822	2.624	2.622	2.821	3.229	0.999	1.145	0.991	0.047
bbg	3.097	3.225	3.392	3.877	1.041	1.143	0.563	0.041
CG42322	2.431	2.457	2.816	3.217	1.011	1.143	0.696	0.049
CG12814	2.540	2.563	2.617	2.989	1.009	1.142	0.756	0.015
Pgant35A	6.452	6.358	4.737	5.404	0.985	1.141	0.538	0.018
nvy	5.190	5.446	6.153	7.012	1.049	1.140	0.077	0.003
CG15436	7.419	7.584	2.983	3.393	1.022	1.138	0.096	0.034
CG5948	2.291	2.293	2.347	2.668	1.001	1.136	0.836	0.012
beat-IV	4.006	4.078	4.654	5.288	1.018	1.136	0.599	0.048
Hsp26	13.039	13.164	7.134	8.098	1.010	1.135	0.434	0.007
CG13067	8.624	9.107	8.883	10.071	1.056	1.134	0.006	0.000
CG10176	6.661	6.861	7.307	8.276	1.030	1.133	0.469	0.003
CG14681	3.166	3.197	3.323	3.763	1.010	1.132	0.686	0.026
Sirt7	7.845	7.860	5.040	5.698	1.002	1.131	0.842	0.014
CG5376	4.385	4.454	4.609	5.205	1.016	1.129	0.106	0.012
CG6621	8.077	8.031	6.374	7.195	0.994	1.129	0.744	0.005
CG9437	6.950	7.077	5.300	5.981	1.018	1.128	0.376	0.044
CG12858	6.571	6.492	6.966	7.860	0.988	1.128	0.462	0.020
CG7300	5.613	5.808	6.642	7.481	1.035	1.126	0.136	0.005
CG30043	5.094	4.172	5.808	6.507	0.819	1.120	0.015	0.078
CG13908	5.538	5.921	6.325	7.077	1.069	1.119	0.030	0.050
Fsh	6.781	6.582	7.078	7.899	0.971	1.116	0.297	0.006
CG15021	6.903	7.401	7.836	8.736	1.072	1.115	0.046	0.010
Lim3	4.795	5.085	5.726	6.381	1.061	1.114	0.138	0.005
pasha	7.374	7.210	5.515	6.142	0.978	1.114	0.164	0.012
Nhe1	9.016	9.835	9.240	10.288	1.091	1.113	0.014	0.000
mol	9.940	10.404	7.397	8.229	1.047	1.112	0.152	0.014
DnaJ-1	12.616	12.421	10.939	12.165	0.985	1.112	0.099	0.003
CG31475	2.181	2.182	2.182	2.425	1.001	1.112	0.225	0.049
CG14798	5.945	5.071	3.185	3.538	0.853	1.111	0.028	0.231
CG18417	5.496	5.932	7.125	7.905	1.079	1.110	0.034	0.024
CG11211	9.083	9.764	9.710	10.774	1.075	1.109	0.018	0.042
CG13299	10.701	9.673	8.163	9.050	0.904	1.109	0.043	0.008
CG11695	5.759	5.810	4.835	5.357	1.009	1.108	0.708	0.012
CG9967	6.165	6.430	4.900	5.424	1.043	1.107	0.181	0.049
CG9062	9.406	9.596	6.440	7.126	1.020	1.107	0.074	0.031
CG17738	2.205	2.181	2.268	2.508	0.989	1.106	0.423	0.005
CG32195	7.431	7.981	8.739	9.664	1.074	1.106	0.006	0.001

Table S3

CG5375	2.883	3.010	3.509	3.880	1.044	1.106	0.551	0.010
CG10344	8.035	7.929	5.506	6.089	0.987	1.106	0.575	0.037
CG4115	6.251	4.584	5.730	6.335	0.733	1.106	0.041	0.003
Cyp313b1	2.284	2.314	2.314	2.555	1.013	1.104	0.184	0.013
CG10420	8.589	8.460	7.023	7.749	0.985	1.103	0.424	0.035
TepIII	6.069	6.181	6.970	7.689	1.019	1.103	0.254	0.013
comm	5.124	5.234	5.683	6.261	1.021	1.102	0.370	0.003
l(2)05714	9.267	9.101	6.906	7.607	0.982	1.102	0.052	0.002
Genes induced by JH in Ovo^{D1} (sterile) but not in fertile females								
	**Normalized mRNA abundance**				**Fold change**		***p*-value**	
9**Gene Symbol**	**wildtype**	**CAKO**	**OvoD**	**OvoD;CAKO**	**wildtype v. CAKO**	**OvoD v. OvoD;CAKO**	**wildtype v. CAKO**	**OvoD v. OvoD;CAKO**
CG13403	2.411	2.480	7.464	3.380	1.029	2.208	0.686	0.000
CG3635	2.364	2.319	5.328	2.433	0.981	2.190	0.232	0.001
Cyp4d21	2.348	2.264	7.876	3.737	0.964	2.108	0.154	0.002
CG30479	2.227	2.231	4.779	2.363	1.002	2.022	0.435	0.012
Lip2	2.166	2.153	4.370	2.240	0.994	1.951	0.309	0.003
UGP	2.931	3.000	5.852	3.051	1.024	1.918	0.422	0.007
CG4835	2.138	2.134	4.091	2.134	0.998	1.917	0.423	0.009
CG5157	2.437	2.280	6.191	3.248	0.935	1.906	0.198	0.016
CG9766	2.168	2.176	4.529	2.392	1.004	1.893	0.624	0.042
CG7763	2.148	2.406	4.405	2.465	1.120	1.787	0.419	0.033
Hr38	2.161	2.160	3.728	2.168	1.000	1.720	0.724	0.033
CG9826	2.208	2.170	3.422	2.243	0.983	1.525	0.096	0.043
Ugt37a1	2.504	2.495	5.436	3.650	0.996	1.489	0.951	0.031
CG34290	2.596	2.439	4.564	3.077	0.939	1.483	0.143	0.019
nimC2	2.152	2.163	3.197	2.184	1.005	1.464	0.359	0.021
Vm34Ca	14.602	13.417	8.118	5.554	0.919	1.462	0.029	0.002
CG17949	11.249	11.828	7.172	4.967	1.051	1.444	0.056	0.002
CG18190	11.149	11.238	4.563	3.244	1.008	1.407	0.442	0.035
CG14946	3.213	3.124	5.773	4.148	0.972	1.392	0.646	0.008
CG30017	2.832	2.725	4.834	3.566	0.962	1.356	0.529	0.028
CG17477	3.498	3.391	5.276	3.914	0.969	1.348	0.034	0.008
alphaTub67C	12.881	12.803	6.217	4.620	0.994	1.346	0.396	0.025
CG9981	2.242	2.733	4.149	3.088	1.219	1.344	0.002	0.063
Ndae1	2.287	2.310	3.277	2.446	1.010	1.340	0.071	0.045
CG1090	2.242	2.217	3.174	2.391	0.989	1.327	0.618	0.046
CG17018	12.604	12.224	6.926	5.253	0.970	1.319	0.005	0.002
Kap-alpha3	5.671	5.379	5.343	4.126	0.949	1.295	0.061	0.002
GRHR	4.355	4.170	5.875	4.546	0.958	1.293	0.244	0.009
Cyp6g2	2.143	2.143	2.763	2.143	1.000	1.289	NA	0.048
CG31445	3.480	3.262	6.883	5.431	0.937	1.267	0.106	0.000
CG3999	3.627	3.363	5.720	4.517	0.927	1.266	0.325	0.020

Table S3

CG14457	4.009	4.202	6.692	5.289	1.048	1.265	0.402	0.009
CG17364	2.609	2.595	3.274	2.609	0.995	1.255	0.809	0.013
CG7422	2.985	2.984	4.497	3.584	1.000	1.255	0.995	0.033
CG8589	8.914	8.900	4.341	3.467	0.998	1.252	0.922	0.026
CG32626	2.287	2.321	3.338	2.668	1.015	1.251	0.641	0.013
CG14544	7.042	6.985	4.148	3.329	0.992	1.246	0.726	0.032
hts	9.720	9.349	5.315	4.279	0.962	1.242	0.010	0.028
GstD10	3.341	3.313	7.136	5.758	0.992	1.239	0.280	0.011
CG10073	2.135	2.134	2.640	2.135	1.000	1.237	NA	0.017
CG18446	10.312	10.809	6.400	5.176	1.048	1.237	0.003	0.049
DNApol-delta	7.434	7.455	4.176	3.380	1.003	1.235	0.865	0.031
tor	8.099	8.068	4.588	3.783	0.996	1.213	0.780	0.019
CG15879	3.092	2.834	4.195	3.489	0.916	1.202	0.060	0.043
l(1)G0196	3.910	3.847	2.669	2.225	0.984	1.200	0.851	0.009
CG4688	4.992	4.749	6.318	5.271	0.951	1.199	0.268	0.025
CG6129	6.716	6.150	8.828	7.442	0.916	1.186	0.082	0.001
Cyp6a8	6.178	5.943	8.522	7.200	0.962	1.184	0.409	0.009
nos	12.901	13.030	5.717	4.835	1.010	1.182	0.483	0.036
CG4500	3.082	5.757	7.953	6.729	1.868	1.182	0.002	0.048
CG32984	7.400	7.019	9.740	8.291	0.949	1.175	0.103	0.000
zpg	11.740	11.841	7.608	6.477	1.009	1.175	0.092	0.023
CG11459	4.415	4.278	5.866	4.999	0.969	1.173	0.766	0.012
Tango13	5.210	5.566	6.494	5.535	1.068	1.173	0.203	0.000
CG3857	7.454	8.248	11.153	9.514	1.107	1.172	0.006	0.001
CG7384	9.841	9.607	7.973	6.831	0.976	1.167	0.005	0.047
Gen	4.102	5.006	2.968	2.544	1.221	1.167	0.033	0.009
CG2678	8.853	8.542	6.216	5.362	0.965	1.159	0.071	0.021
CG17322	8.591	7.911	8.551	7.377	0.921	1.159	0.042	0.003
CG7433	2.396	2.400	2.824	2.457	1.002	1.149	0.401	0.026
homer	7.530	7.452	6.036	5.253	0.990	1.149	0.629	0.027
CG8299	7.466	7.503	10.445	9.106	1.005	1.147	0.920	0.001
CG7714	2.262	2.187	2.713	2.368	0.967	1.146	0.042	0.006
asparagine-synthetase	9.254	8.935	10.106	8.845	0.966	1.143	0.031	0.013
CG9925	10.789	10.666	7.592	6.659	0.989	1.140	0.292	0.048
phr	7.785	7.321	6.897	6.056	0.940	1.139	0.101	0.013
CG7191	3.111	3.125	4.734	4.162	1.004	1.138	0.696	0.049
CG6687	6.801	6.506	9.711	8.537	0.957	1.137	0.315	0.015
wdb	5.118	5.831	3.178	2.806	1.139	1.133	0.127	0.028
pk	3.259	3.056	4.249	3.756	0.938	1.131	0.074	0.026
Snoo	7.394	7.139	9.272	8.195	0.965	1.131	0.150	0.004
CG10361	4.302	4.359	7.316	6.470	1.013	1.131	0.724	0.003
CG31611	13.811	14.240	7.878	6.978	1.031	1.129	0.119	0.004

Table S3

nord	7.133	6.527	8.041	7.126	0.915	1.128	0.011	0.010
CG13776	4.559	4.574	3.987	3.534	1.003	1.128	0.904	0.027
yellow-f	3.178	3.889	5.594	4.960	1.224	1.128	0.060	0.047
CG32154	2.449	2.341	2.990	2.651	0.956	1.128	0.406	0.023
CG6912	6.966	6.493	9.719	8.630	0.932	1.126	0.256	0.003
Btd	2.176	2.176	2.474	2.197	1.000	1.126	0.423	0.012
pch2	8.831	8.821	6.565	5.836	0.999	1.125	0.933	0.008
CG10006	4.245	4.585	7.130	6.346	1.080	1.124	0.430	0.015
CG32698	5.335	5.556	6.587	5.868	1.041	1.123	0.080	0.006
Sox102F	5.936	5.433	7.394	6.588	0.915	1.122	0.116	0.021
CG8611	7.893	7.650	6.303	5.616	0.969	1.122	0.015	0.025
Nmdmc	10.315	9.669	10.227	9.117	0.937	1.122	0.034	0.022
fru	2.468	2.407	2.612	2.330	0.975	1.121	0.561	0.001
CG1946	5.768	6.415	8.504	7.591	1.112	1.120	0.104	0.023
CG3335	8.040	7.790	6.689	5.972	0.969	1.120	0.053	0.040
Atf-2	5.132	5.294	6.711	6.006	1.032	1.117	0.463	0.016
yl	11.444	11.092	4.245	3.800	0.969	1.117	0.033	0.036
CG18278	8.155	7.984	6.974	6.246	0.979	1.117	0.252	0.027
CG40178	6.394	6.403	7.356	6.593	1.002	1.116	0.957	0.002
CG9344	9.243	8.726	8.258	7.407	0.944	1.115	0.050	0.006
to	9.545	9.185	12.193	10.940	0.962	1.114	0.014	0.008
CG7922	6.850	6.567	6.278	5.650	0.959	1.111	0.017	0.023
CG8607	7.524	7.399	5.788	5.210	0.983	1.111	0.215	0.004
Ptp99A	7.361	6.996	6.372	5.738	0.950	1.110	0.107	0.026
mbm	10.113	9.981	7.320	6.594	0.987	1.110	0.096	0.002
RpS30	8.447	8.280	7.573	6.825	0.980	1.110	0.036	0.027
CG31809	7.465	6.910	9.569	8.631	0.926	1.109	0.016	0.015
CG12560	7.625	7.597	9.245	8.342	0.996	1.108	0.852	0.001
Cyp6a20	7.185	7.872	10.388	9.375	1.096	1.108	0.007	0.001
CG4725	4.314	4.126	6.287	5.677	0.956	1.107	0.361	0.017
Papss	7.797	7.319	7.961	7.188	0.939	1.107	0.031	0.003
CG12116	13.137	12.259	13.135	11.882	0.933	1.105	0.014	0.002
CG10341	8.681	8.371	8.200	7.426	0.964	1.104	0.001	0.014
Oatp30B	9.393	8.987	9.862	8.941	0.957	1.103	0.019	0.000
CG32155	5.554	5.505	7.207	6.536	0.991	1.103	0.880	0.014
CG5151	8.042	7.874	8.390	7.610	0.979	1.103	0.098	0.020
Su(var)2-HP2	7.994	7.831	6.098	5.534	0.980	1.102	0.283	0.027
M(2)21AB	14.055	13.228	13.576	12.321	0.941	1.102	0.000	0.000
bam	8.636	8.719	2.843	2.582	1.010	1.101	0.557	0.027

Table S3

