# Supplementary material for: Juvenile hormone regulation of Drosophila aging
Source: BMC Biol. 2013 Jul 17;11:85. doi: 10.1186/1741-7007-11-85 (PMC3726347; doi:10.1186/1741-7007-11-85)
Supplement: Additional file 6: Table S4 — Primers for qPCR. [file 1741-7007-11-85-S6.pdf]

Primer Name	Forward or Reverse 5'-3' sequence
DmKr-h1-comm-f	TGCCAGAAAGCACTGTGGACTACT
DmKr-h1-comm-r	AGCAGGGCAGGATTGATGGTTAGA
DmJon25Bii-f	AGCACTCGGACTACAACACCAACA
DmJon25Bii-r	TGTTGATCAGGTGCCAGAAGTCCA
takeout-f	ATGAAGCTGTGCAACACCCTGTTC
takeout-r	ACGATCCTTTGGTCCTTGATCCCA
obp99b-f	AGCACGGATTCGATGTCCACAAGA
obp99b-r	TTGGAGTTCATGAAGCACATGCCG
ana-f	GGCGAGTTGGACATTGCCAAGAAA
ana-r	TTGTCGATCGTGGGTGAGTTGGAT
yp1 - f	TCCAAGCTGAACACCTATGAGCGT
yp1 - r	TGGTATCGAAGGGCATGTCCAACT
yp2 - f	AGGAAGCAGAACGGTGAACAGGAT
yp2 - r	TACTGCTCAAAGTCCTCGATGGCA
yp3 - f	TACCACGTTGGCCAAATCAAGCAC
yp3 - r	TGGCCGTCTCCACATAGTTGTTCA
lsp1 alpha - f	CAACATGCTCAACGGCAAGATGGT
lsp1 alpha - r	TCATCGAACTTGGGTCCCAGGAAA
fbp1 - f	AGAATGGACGCAACCGCATTCATC
fbp1 - r	CAATTGCAGCATCACTTGGCGGTA
dlp2 -f	CCCTGCAGTTTGTCCAGGAG
dlp2 -r	CCAGGAAAGAGGGCACTTCG
dlp3 -f	AAGACCGTTCCCTGCTGGAA
dlp3 -r	CCATCCCGAAGACGTCGAGT
dlp6 -f	CCCTTGGCGATGTATTTCCCAACA
dlp6 -r	CCGACTTGCAGCACAAATCGGTTA
dlp5 -f	CAATTCAATGTTCGCCAAACG
dlp5 -r	GGAGCTATCCAAATCCGCCA
